# Serine Protease Inhibitor Kazal Type 1, A Potential Biomarker for the Early Detection, Targeting, and Prediction of Response to Immune Checkpoint Blockade Therapies in Hepatocellular Carcinoma

**DOI:** 10.3389/fimmu.2022.923031

**Published:** 2022-07-18

**Authors:** Jianlong Jia, Latai Ga, Yang Liu, Zhiyi Yang, Yue Wang, Xuanze Guo, Ruichen Ma, Ruonan Liu, Tianyou Li, Zeyao Tang, Jun Wang

**Affiliations:** ^1^ Department of Pathophysiology, College of Basic Medical Sciences, Dalian Medical University, Dalian, China; ^2^ Department of Pharmacology, College of Pharmacy, Dalian Medical University, Dalian, China

**Keywords:** serine peptidase inhibitor Kazal type 1 (*SPINK1*), immune checkpoint blockade (ICB), hepatocellular carcinoma (HCC), tumor immune microenvironment (TIME), biomarker

## Abstract

**Background:**

We aimed to characterize serine protease inhibitor Kazal type 1 (*SPINK1*) as a gene signature for the early diagnosis, molecular targeting, and prediction of immune checkpoint blockade (ICB) treatment response of hepatocellular carcinoma (HCC).

**Methods:**

The transcriptomics, proteomics, and phenotypic analyses were performed separately or in combination.

**Results:**

We obtained the following findings on *SPINK1*. Firstly, in the transcriptomic training dataset, which included 279 stage I and II tumor samples (out of 1,884 stage I–IV HCC specimens) and 259 normal samples, significantly higher area under curve (AUC) values and increased integrated discrimination improvement (IDI) and net reclassification improvement (NRI) were demonstrated for HCC discrimination in *SPINK1*-associated models compared with those of alpha-fetoprotein (AFP). The calibration of both *SPINK1*-related curves fitted significantly better than that of AFP. In the two independent transcriptomic validation datasets, which included 201, 103 stage I-II tumor and 192, 169 paired non-tumor specimens, respectively, the obtained results were consistent with the above-described findings. In the proteomic training dataset, which included 98 stage I and II tumor and 165 normal tissue samples, the analyses also revealed better AUCs and increased IDI and NRI in the aforementioned *SPINK1*-associated settings. A moderate calibration was shown for both *SPINK1*-associated models relative to the poor results of AFP. Secondly, in the *in vitro* and/or *in vivo* murine models, the wet-lab experiments demonstrated that *SPINK1* promoted the proliferation, clonal formation, migration, chemoresistance, anti-apoptosis, tumorigenesis, and metastasis of HCC cells, while the anti-*SPINK1* antibody inhibited the growth of the cells, suggesting that *SPINK1* has “tumor marker” and “targetable” characteristics in the management of HCC. The Gene Ontology (GO) and Kyoto Encyclopedia of Genes and Genomes (KEGG) analyses revealed that *SPINK1* was engaged in immunity-related pathways, including T-cell activation. Thirdly, in the transcriptomic analyses of the 368 HCC specimens from The Cancer Genome Atlas (TCGA) cohort, the high abundance of *SPINK1* was positively correlated with the high levels of activated tumor-infiltrating CD4^+^ and CD8^+^ T lymphocytes and dendritic and natural killer cells, while there were also positive correlations between *SPINK1* and immune checkpoints, including PD-1, LAG-3, TIM-3, TIGIT, HAVCR2, and CTLA-4. The ESTIMATE algorithm calculated positive correlations between *SPINK1* and the immune and ESTIMATE scores, suggesting a close correlation between *SPINK1* and the immunogenic microenvironment within HCC tissues, which may possibly help in predicting the response of patients to ICB therapy.

**Conclusions:**

*SPINK1* could be a potential biomarker for the early detection, targeted therapy, and prediction of ICB treatment response in the management of HCC.

## Introduction

With a dismal prognosis, hepatocellular carcinoma (HCC) has 5-year recurrence rates approaching 70% post-resection, and overall survival (OS) is about 3–4 months in patients with untreated HCC (1). Due to the complexity of tumor heterogeneity (2–4), the three current main obstacles in the management of HCC are mining optimal biomarkers for its early diagnosis, molecular targeted therapeutics, and prediction of the efficacy of immune checkpoint blockade (ICB) therapies. Although exploration of some biomarkers has led to new surveillance programs for the early detection of HCC, the pros and cons of these molecules need to be reevaluated and improved (2, 3), as almost half of patients are still in the advanced stage at the time of diagnosis (5). As is known, over the past decade, targeted therapy has become one systemic therapy option for patients with HCC, despite the fact that only ~25% of HCCs harbor alterations that are potentially targetable with existing drugs (6). But these treatments have shown limited efficacy, and more reliable molecular targets are required for therapeutics through further understanding of the pathogenesis of HCC (7). On the other hand, ICB therapies have become increasingly important since tumor cells and infiltrating immune cells harbor an immunosuppressive microenvironment, including the overexpression of immune checkpoint molecules (8). However, ICB monotherapy has a response rate of only about 15%–23%, while that of combination treatments increased to approximately 30% (9). In addition, ICBs can induce severe immune-related side effects (10, 11). Therefore, it is necessary to use more reliable biomarkers for the prediction of the efficacy of ICB prior to starting a treatment plan. Unfortunately, based on our literature search, there are no predictive biomarkers currently available for the prediction of ICB response (5). In addition, it is costly and inconvenient for patients to undergo gene sequencing screening before deciding on ICB and targeted therapeutics. Thus, the discovery of potentially actionable biomarkers is necessary for the early diagnosis, target therapy, and prediction of ICB response to immunotherapy of HCC.

Combining biostatistical analyses and wet-lab experiments, the aim of this study was to explore *SPINK1*, a potentially qualified candidate, as a better marker for the early diagnosis, molecular targeting, and the prediction of the response to ICB treatment of HCC.

## Materials and methods

### Online Transcriptomics Studies of Liver Cancer Cohorts

The preliminary complementary DNA (cDNA) expression of *SPINK1* in HCC tissue samples was analyzed using Oncomine (12). Gene expression analyses were carried out using “Oncomine” on four cohorts of patients with HCC, including Roessler Liver 1 and 2, Chen Liver, and Wurmbach Liver. The expression levels of *SPINK1* messenger RNA (mRNA) were assessed using liver tumor and precancerous tissues, and differences with *p* ˂ 0.05 were considered statistically significant. Oncomine is publicly available at www.oncomine.org.

### UALCAN, HPA Data Sources, and Kaplan–Meier Plotter

UALCAN is an easy-to-use, interactive web portal for conducting in-depth analyses of The Cancer Genome Atlas (TCGA) gene expression data (13). HPA, the Human Protein Atlas project, is funded to generate a map of protein expression patterns in normal cells, tissues, and cancer, while the Kaplan–Meier plotter is capable of assessing the impact of genes on the survival of many cancer types.

UALCAN uses TCGA level 3 RNA sequencing (RNA-seq) and clinical data from 31 cancer types including HCC. This web portal allows analysis of the relative expression of a query gene across liver tumor and normal tissue samples, as well as in various tumor subgroups including individual cancer stages, grade, race, body weight, or other clinicopathological features. UALCAN is publicly accessible at http://ualcan.path.uab.edu. The expression levels of some specific proteins corresponding to over 50% of all human protein-encoding genes have been analyzed and can be obtained from the HPA website. The HPA-related dataset is available at https://www.proteinatlas.org. The Kaplan–Meier plotter evaluates the impact of genes on the clinical outcomes of patients with different cancer types. Sources for the databases include the Gene Expression Omnibus (GEO) and TCGA. These two databases are freely available at http://gepia.cancer-pku.cn and http://kmplot.com/analysis.

### Bioinformatics Analysis of the GEO and RNA-Seq Datasets

For the early detection of HCC, out of 1,884 specimens of stage I–IV HCC, 538 stage I and II patient samples (279 tumor and 259 para-tumor samples) from GEO (GSE14520, GSE101685, and GSE62232) were selected and used as the training dataset. The two new, non-mixed cohorts of 395 (GSE36376) and 272 (OEZ005255, NODE database) stage I and II specimens (201 tumor, 192 para-tumor; 169 tumor and 103, para-tumor) from the GEO RNA-seq datasets, respectively, were used as the validation datasets (14, 15). The list of NCBI-GEO datasets employed to perform the initial biomarker screening included GSE102097, GSE107170, GSE143231, GSE14520, GSE22405, GSE29721, GSE31370, GSE39791, GSE41804, GSE45267, GSE46408, GSE51401, GSE54236, GSE57957, GSE62232, GSE69715, GSE76427, GSE84402, GSE84598, GSE89377, and GSE98383 (**Supplementary Table S1**). The RNA-seq data collection used for the analysis of predictive factors was from Gao et al. (15) (OEZ005255, NODE database). All of the datasets comprise 1,884 tumor and non-tumor normal tissue samples from patients with stage I–IV HCC. Out of the aforementioned sample pool, three HCC datasets were collected from the GEO database (GSE14520, GSE101685, and GSE62232). These datasets included a total of 611 samples, of which 538 stage I and II patient samples were chosen as the training dataset. The TNM staging system of the American Joint Committee on Cancer (AJCC) was used to classify the stage I and II patient specimens from the rest of the samples. The original expression profiling data were obtained from both cancerous and non-cancerous tissue specimens. For the microarray probes, the expression values for the probe-mapped genes were calculated to obtain the means of the probes if multiple probes were mapped to the same Entrez gene ID. Data batch effects among the GEO data files were examined with *t*-distributed stochastic neighbor embedding (tSNE) analysis and corrected using ComBat of the R package “sva.” (14)

The RNA-seq data derived from 272 liver tissue specimens and para-cancerous normal tissue samples of patients with HCC could be obtained either from downloading the files from the website (https://www.biosino.org/node/index) or directly from the authors who published it. This transcriptomics dataset from Gao et al. (15) is publicly available to researchers.

For the analyses of the prediction of response to ICB therapy, 368 stage I–IV HCC specimens were selected from TCGA RNA-seq database (https://xenabrowser.net/datapages/), as the other GEO and RNA-seq training and validation datasets lack the transcriptomic abundance information on the expression of programmed death-ligand 1 (PD-L1), which was needed for the analysis of the biomarker-related ICB response.

### Proteomics Analysis

Paired 98 tumor and 165 para-tumor tissue samples from stage I and II Chinese HCC patients (CHCC) (out of 330 I–IV tumor samples) with hepatitis B virus (HBV) infection were selected for proteomics analysis (15). The published HCC proteomics CPTAC (the National Cancer Institute’s Clinical Proteomic Tumor Analysis Consortium) data file was obtained directly from the web link provided by Professor Hu Zhou, University of Chinese Academy of Sciences, Beijing (https://cptac-data-portal.georgetown.edu/study-summary/S049). In the proteomics analysis, 10,783 proteins (encoded by 10,759 genes) with an average of 8,934 proteins per sample were identified using isobaric tandem mass tag (TMT)-based global proteomics (15).

### Cell Culture, Vector, Antibodies, and Reagents

The HepG2, Hep3B, H22, and 293T cell lines were given as gifts by Professor Aiguo Wang (Dalian Medical University, Dalian, China), who purchased them from the Chinese Academy of Sciences, Beijing, China. The cells were grown in McCoy’s medium (Sangon Biotech, Shanghai, China), RPMI-1640, or Dulbecco’s modified Eagle’s medium (DMEM) (Sangon Biotech, Shanghai, China) with 10% fetal bovine serum (FBS) (CellMax, AusGeneX, Queensland, Australia). Human *SPINK1*-overexpressing (OE) and *SPINK1* short hairpin RNA (shRNA) lentiviral vectors were generated by Applied Biological Materials (ABM, Nanjing, China), and the Transwell system was purchased from Nest (Southborough, MA, USA). The following commercially available antibodies were used: anti-SPINK1 (Abnova, Taipei, Taiwan) and anti-Bcl-2, anti-Bcl-XL, anti-Bax, anti-Bad, and anti-caspase-3 (all from Wanlei Biotech, Shenyang, China). The main chemicals or reagents used in this study were as follows: 5-fluoruracil (5-FU) (Shanghai Pharmaceutical Company, Shanghai, China), Lipo2000 (Invitrogen, Carlsbad, CA, USA), Cell Counting Kit-8 (CCK-8) (Xian Baiying Biotechnology Inc., Xian, China), puromycin (Solarbio, Beijing, China), and polybrene (Maokang Biotech, Shanghai, China).

### Generation of Stable *SPINK1*-Overexpressing or *SPINK1*-Silencing HCC Cell Lines

The HepG1, Hep3B, and H22 HCC tumor cell lines permanently transduced with *SPINK1*-OE or *SPINK1*-silencing (shRNA) lentiviral constructs were generally constructed as previously described (16). Briefly, the pLenti-CMV-hSPINK1-2A-GFP vector or pLenti-SPINK1 shRNA was co-transfected with either ABM’s Second Generation (LV003) Packaging Mix or the mixture of pMD2.G and psPAX2 packaging plasmids (Addgene Inc., Cambridge, MA, USA) into 293T cells, while the transfected empty or scrambled vector was used as a control. The packaged lentiviral particles were harvested 24–48 h after transfection. HepG2, Hep3B, and H22 cells were infected by filtered lentiviral particles, and the transduced cells were screened under pressure of 2 µg/ml puromycin for about 3 weeks before the drug-resistant cells were collected. Subsequently, the protein expression of *SPINK1* was examined in passaged HepG1, Hep3B, and H22 cells using Western blotting.

### Proliferation, Colony Formation, Transwell Migration, Scratch Test (Wound Healing Assay), Chemoresistance, Anti-Apoptosis, and *SPINK1* Targeting Response in HCC Cells

Experiments were carried out with respect to colony formation, Transwell assay, chemoresistance, chemically induced apoptosis resistance, and *SPINK1* targeting response in *SPINK1*-OE and/or knockdown HCC cells. Cell proliferation was measured using CCK-8, as described in a previous publication (16). In brief, the experiments were performed in cell culture dishes or in triplicates of a 96-well plate for each designed group. The absorbance (*A*) for the wells was measured using a microplate reader. The proliferation rates were calculated as: inhibition rate (%) = (1 − treated group *A*/control *A*) × 100%. The colony formation assay was carried out as follows. Briefly, the corresponding HCC cells were seeded in six-well plates at about 1,000 cells per well and cultured for 14 days. The plates were then washed with phosphate-buffered saline (PBS) and stained with crystal violet. The images of each well were saved, and the number of holoclones re-grown (colonies >50 cells each) was counted to determine the efficacy of holoclone formation. The clone formation rate was equal to the number of clones/cells seeded (in percent). The experiment was performed in triplicate.

*In vitro* Transwell migration assays were performed using Nest 12-multiwell inserts with 8-µm pores. For the migration assay, 30,000 cells in 200 µl serum-free DMEM were placed into each Transwell insert with 750 µl of 10% FBS growth medium in the lower chamber. The cells migrated through the pores were processed with 3.7% formaldehyde, stained with 2.5 µg/ml Hoechst for 15 min before washing with PBS, and counted 48 h after cell seeding. The 10 fields of cells were observed per experiment.

For the scratch test, 5 × 10 (4) cells/well were seeded into six-well plates and cultured to the second day. Three parallel marks were scratched along the margin of a ruler in each well using a 200-μl pipette tip. With the supernatant removed, serum-free medium was added to the cells and the cells photographed under a microscope after 0, 24, and 48 h. The migration rate of the cells in each group was calculated with ImageJ software.

For the chemoresistance test, *SPINK1-*OE and/or knockdown HepG2, H22, or Hep3B cells with or without 5-FU (2.5 μM) treatment were inoculated in a 96-well plate (0.3 × 10 (4)/well) for 48 h before 10 μl of CCK-8 solution was finally added into each well; the incubation was continued for an additional 2 h prior to the analysis. The apoptosis status of *SPINK1*-OE or knockdown HCC cells with or without 5-FU treatment was evaluated by examining a number of the main pro- and anti-apoptotic molecules using Western blotting. After seeding with 0.3 × 10 (4) cells/well, the *SPINK1* targeting experiment was carried out by assessing the targeting efficacy of the human anti-*SPINK1* antibody in HCC cells at a concentration of 5 or 10 μg/ml using the CCK-8 assay after 72 h culture in a 96-well plate.

### Conditioned Media and Rescue Experiment

For the collection of conditioned medium (CM), 8–10 ml of serum-free DMEM was added into each of a 10-cm culture plate seeded with HepG2 and the *SPINK1*-OE cell lines. The cell debris was removed and *SPINK1* CM was collected 48 h later *via* centrifugation at 1,000 rpm for 5 min at 4°C. The supernatant was accumulated and stored at −80°C until use. The vector control CM was made for the empty vector-transfected cell strains in the same manner as described above. For the rescue experiment, permanent *SPINK1*-silencing Hep3B and empty vector-transduced control cells were treated with *SPINK1* CM and control CM, respectively, for 48 h. Subsequently, the CCK-8 assay was performed to evaluate the proliferation rates of these two cell lines processed with the CM.

### Western Blotting

The immunoblot analysis was performed as previously described (17). For this analysis, whole cell lysates were prepared using NP-40 lysis buffer, followed by measurement of the protein concentration. The proteins were separated by SDS-PAGE and transferred to a 0.2-mm PVDF membrane. SPINK1, GAPDH, and other antibodies including Bcl-2, Bcl-XL, Bid, and Bad were used to probe the targeted protein at 1:500 before the antibody–antigen complex was detected using horseradish peroxidase (HRP)-conjugated antibodies and enhanced chemiluminescence (ECL).

### Experiments of Tumor Homograft, Xenograft, and Metastasis *In Vivo*

*SPINK1*-associated tumorigenesis and metastasis were analyzed in normal immunocompetent BALB/c mice using *SPINK1*-OE HepG2 or H22 cells. Clean-degree female BALB/c mice (6–8 weeks old, weighing around 20 g) were purchased from the Center of Experimental Animals of Dalian Medical University. The mice were reared in a room on a 12-h light/dark cycle. All procedures were approved by the Animal Care and Use Committee of Dalian Medical University. The tumor volume and metastatic lung nodules were measured based on the methods in previously published reports (18, 19). For the growth study, 3 × 10 (6) cells/0.2 ml scramble control and SPINK1–OE HepG2 or H22 cells were injected subcutaneously in the back region of the mice. Tumor growth was recorded weekly using a caliber ruler, and the tumor volume was estimated using the formula: [tumor volume = 1/2(L × W (2)]. The tumor burden or weight was scaled as soon as the mouse was sacrificed. For the metastasis study, 3 × 10 (5) cells/0.05 ml scramble control and SPINK1–OE H22 cells were injected *via* the tail vein, and the lungs of mice were simultaneously excised and observed grossly for metastatic lesions through counting the total volume of the number of metastatic nodules using the same method described above: tumor volume = 1/2[L × W (2)].

### Co-Expression, Enrichment, and Pathway Analyses of *SPINK1*

LinkedOmics is a publicly available portal that includes multi-omics data from all 32 TCGA cancer types (20). The LinkFinder module of LinkedOmics was used to study the genes co-expressed with *SPINK1* in the HCC cohort of TCGA. The results showed that a large number of genes had significantly positive correlations with *SPINK1*, whereas similar numbers of genes showed significantly negative correlations [false discovery rate (FDR) <0.01]. These data were statistically evaluated using Pearson’s correlation coefficient, followed by graphical presentation in volcano plots and heatmaps. The LinkInterpreter module of LinkedOmics carries out Gene Ontology (GO) and Kyoto Encyclopedia of Genes and Genomes (KEGG) analyses of the genes differentially co-expressed with *SPINK1*. GO and KEGG analyses were performed with the “GSEA” tool. The LinkedOmics database is publicly available at http://linkedomics.org/login.php.

### Potential Predictor of Response to ICB Therapy

In order to predict patients more likely to benefit from ICB immunotherapy (21), we presented the currently recognized immune response-associated cells or molecules and analyzed their correlation with *SPINK1* using their unique expression data, assessing the possibility of *SPINK1* as a predictor for precision HCC immunotherapy.

For the assessment of the association between *SPINK1* and tumor-infiltrating immune cells, the transcriptomic expression of *SPINK1* within each tumor sample of the HCC dataset (TCGA) was initially subcategorized as high or low level on the basis of its median value. Subsequently, the compositions and contents of the various subtypes of infiltrating immune cells in each sample were determined based on RNA expression abundance using CIBERSORT. Thirdly, the correlations between *SPINK1* and the expression patterns of 28 subtypes of infiltrating immune cells were analyzed in the same “high” or “low” expression group of specimens using statistical *t*-test. With respect to the estimation of stromal and infiltrating immune cells in malignant tumor tissues, we chose a new algorithm that takes advantage of the unique properties of the transcriptional profiles of the HCC samples to deduce tumor cellularity, named ESTIMATE (Estimation of Stromal and Immune Cells in Malignant Tumor Tissues Using Expression Data) (22). The correlations between *SPINK1* and the immune, stromal, and ESTIMATE scores were determined using the “estimate” package in R. Similarly, the expressions of several prominent immune checkpoints (ICPs) were determined as these important molecules are likely involved in the induction of ICB response (23). Tumor mutation burden (TMB), the number of mutations per million bases in tumor tissue, was used for assessing vulnerability to ICB immunotherapy, as TMB likely affects immune response (24).. The mutation data of patients with HCC were downloaded from TCGA, and TMB analysis was accomplished using the R package “maftools.” (25) The correlations among *SPINK1*, ICPs, and TMB were investigated using statistical *t*-test.

### Statistical Analysis Software and Methods

Most of the bioinformatics analyses were accomplished using R statistical software, version 3.4.1 (R Core Team, Vienna, Austria). MedCalc software was used to plot the area under the curve (AUC) for AFP, *SPINK1*, and their combination before carrying out logistic regression to generate the receiver operating characteristic (ROC) curves. The “rms” package was used to draw the fit curves of the observed and predicted values. The R package “PredictABEL” was applied to calculate the integrated discrimination improvement (IDI) and net reclassification improvement (NRI), which represent the discrimination of a prediction pattern (26). The Akaike information criterion (AIC) and Bayesian information criterion (BIC), which reveal the calibration of the same model, were also applied (27). We performed decision curve analysis (DCA), a measurement of the clinical net benefits (28), using the “Decision Curve” package in R. The clinical impact curve (CIC) (29), a plot derived from the DCA, was used to further evaluate the clinical cost/benefit ratio of a model. Significant differences in the murine tumor volume among the groups were statistically analyzed using two-way ANOVA. Quantitative or semi-quantitative variables were counted as the mean ± SEM, and the means were compared using unpaired two-tailed Student’s *t*-test. A *p*-value <0.05 was considered statistically significant.

## Results

### Study Design

**Figure 1:**
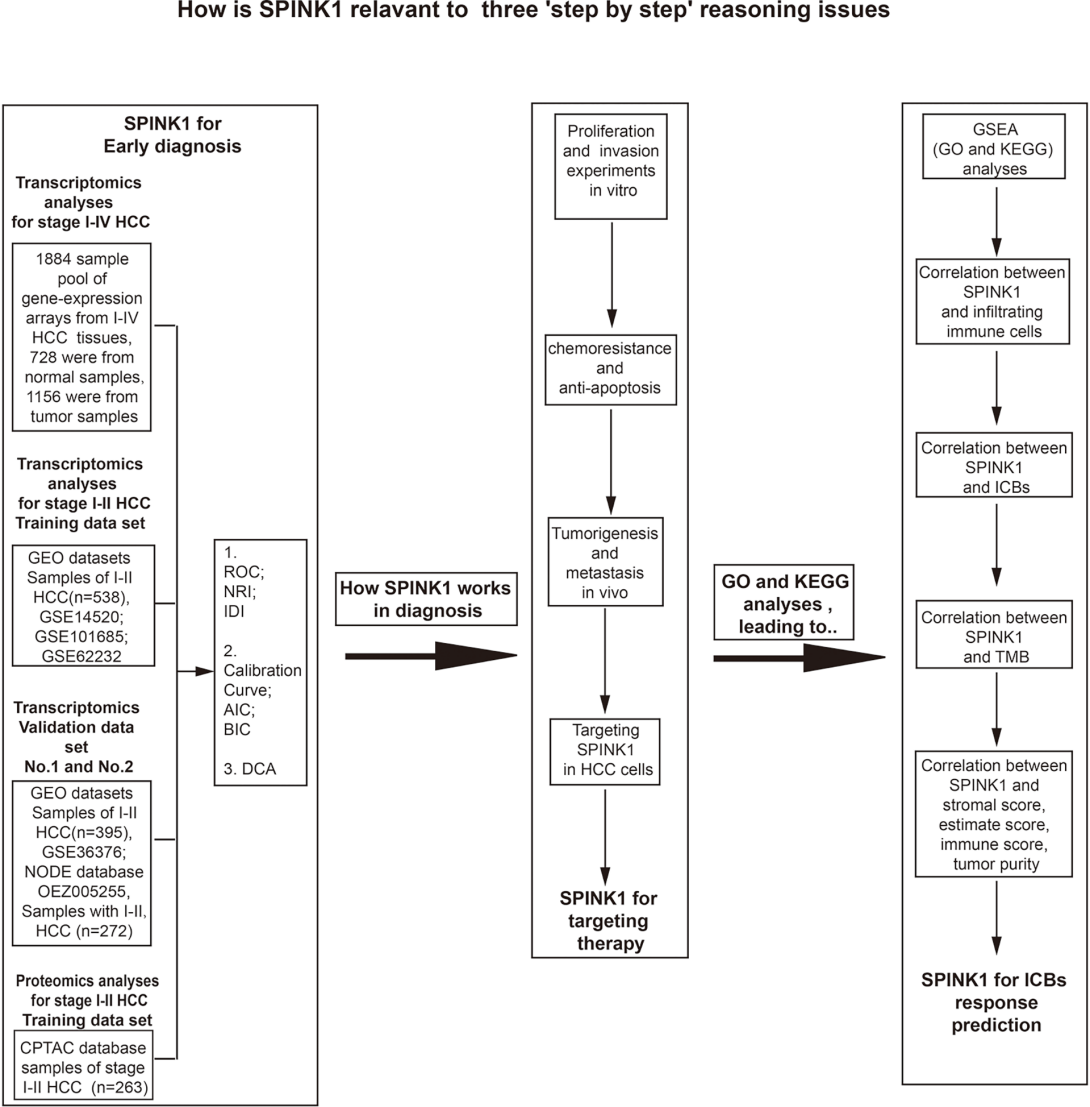
Flowchart of the three goals of this investigation: step-by-step bioinformatics analyses, bench-side experimental design, and eligibility criteria for the collection of tissue samples from patients with hepatocellular carcinoma (HCC) from different datasets.

The flowchart shows the research project process (**Figure 1**). The study design included three step-by-step reasoning concerns we encountered during the investigation, the eligibility criteria for the selection of patients with HCC, and the bench-scale experiment plans during the analysis process (**Figure 1**). In the transcriptomics study of “HCC early detection,” the cDNA microarray expression data files were used in the analysis of the training dataset, while the GEO and RNA-seq data files were applied in two new and non-mixed validation datasets, respectively. The training dataset was used only in the proteomics analysis for the above detection. To collect more evidence of “what works and why” for the role of *SPINK1* in the early detection of HCC, we carried out *in vitro* and *in vivo* wet-lab experiments including monoclonal antibody (mAb)-oriented or molecular targeting and other cellular assays (**Figure 1**). Interestingly, we found that *SPINK1* possesses some biologically tumorigenic characteristics that make it a potential target molecule in targeted therapeutics for HCC. Thereafter, we further explored the underlying molecular signaling networks that mainly contributed to the “early detection” and “targeting” features of *SPINK1*. Unexpectedly, gene set enrichment analyses (GSEA) including GO and KEGG showed that the underlying molecular interactions were closely associated with immune response-related biological processes and pathways in HCC. Thus, we assumed that *SPINK1* might have acted as a biomarker for the prediction of response to ICB therapy. In the “prediction” study of ICB-related therapies, the RNA-seq data of the HCC samples from the TCGA database were collected for the analyses of the associations between *SPINK1*, infiltrating immune cells, stromal cells, immune checkpoint molecules, and TMB (**Figure 1**).

### Overexpression of *SPINK1* in HCC Tissues

We initially examined the cDNA expression profiles of *SPINK1* in the liver samples of four cohorts (Roessler Liver 1 and 2, Chen Liver, and Wurmbach Liver) of patients with HCC from Oncomine (30). The mRNA expression of *SPINK1* was significantly higher in the HCC liver tissue samples of three cohorts (Roessler Liver 1 and 2 Chen Liver) compared to normal controls (*p* ˂ 0.01; **Supplementary Figures S1A–D**). In the UALCAN analysis, the transcription level of *SPINK1* was ranked within the top 18 out of all the overexpressed genes in HCC tissues (**Supplementary Figure S2**) and was significantly higher in the HCC tissue specimens of stage I–III compared to stage IV tumor and grades I–IV (*p* < 0.05; **Supplementary Figures S1E,F**). Furthermore, the protein expression of *SPINK1* was significantly increased immunohistochemically in 6 out of the 12 HCC tissue sections, as shown by the HPA data source (**Supplementary Figure S1G**) (31). Clinically, the Kaplan–Meier plotter (32) showed that a high *SPINK1* mRNA expression was significantly associated with poor OS of patients with HCC compared with patients with low mRNA expression (data not shown). In contrast, there was no significant correlation between AFP and OS in HCC patients (data not shown). These above preliminary data suggested a possible role of *SPINK1* as a biomarker for the detection and even early diagnosis of patients with HCC.

### Early Detection of HCC *via* Analyses of cDNA Microarray Expression and RNA-Seq Profiles

We firstly mined the cDNA expression data of the 1,884 human HCC samples, which showed that the *SPINK1* gene was significantly overexpressed in stage I–IV tumor samples relative to para-tumor specimens and presented better discrimination and calibration capabilities for HCC detection than AFP (**Supplementary Figure S3**, **Table S2**). We then chose the stage I and II tumor samples out of the 1,884 and used them as the training dataset for further analyses.

**Figure 2:**
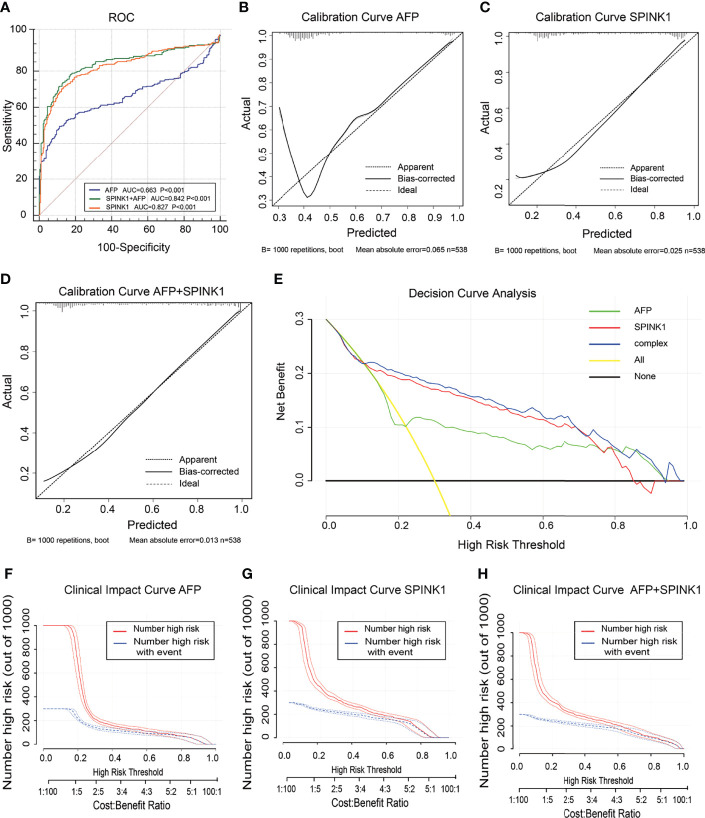
Discrimination and calibration of hepatocellular carcinoma (HCC) prediction models through transcriptomics analyses in the training dataset. **(A)** Receiver operating characteristic (ROC) curves and area under the curve (AUC) in the *SPINK1*, *SPINK1* plus alpha-fetoprotein (AFP), and AFP models. **(B–D)** Calibration curves of AFP **(B)**, *SPINK1* **(C)**, and *SPINK1*+AFP **(D)**. **(E)** Clinical net benefits in the decision curve analysis (DCA) of *SPINK1*, *SPINK1*+AFP, and AFP. *Yellow line* indicates all HCC patients; *middle and horizontal black line* denotes non-HCC patients. **(F–H)** Comparison of the clinical benefits obtained from the clinical impact curve (CIC) analysis of each detection model.

In the training dataset, we found that the ROC curves plotted for *SPINK1* separated the stage I and II HCC samples from the normal adjacent to tumor controls with high discriminatory accuracy (**Figure 2**). Compared with AFP, which lacked sufficient sensitivity in the detection (33, 34), the *SPINK1* and *SPINK1*+AFP models provided higher AUC values with similar specificity and a much higher sensitivity for patients with HCC (AUC = 0.83 vs. 0.84 vs. 0.66, specificity = 87.6% vs. 83.0% vs. 88.4%, sensitivity = 70% vs. 77.8.1% vs. 49.5%) (**Figure 2A**). Notably, the transcription level of *SPINK1* was not correlated with that of AFP, suggesting *SPINK1* as an independent diagnostic factor for HCC (*R* = 0.42; **Supplementary Figure S4**). To further determine how much better a new *SPINK1*-related model reclassified patients into the HCC and non-HCC groups, we calculated the IDI and NRI parameters. The *SPINK1*-related models remarkably improved the classification capacity of AFP (IDI = 118.4%:122.6%:reference, *p* < 0.001) (**Table 1**). Using the cutoff values at 0%–30% (low risk), 30%–60% (moderate risk), and 60%–100% (high risk), the NRI of the two aforementioned *SPINK1* models increased by 71.6% and 79.6%, respectively, compared to that of AFP alone (all *p* < 0.01; **Table 1**), suggesting that *SPINK1* improved the classification of patients with events and those without events. Thus, *SPINK1* has a higher discriminatory capacity than AFP.

**Table 1:** Discrimination and calibration of three predictive models.

	AFP	*SPINK1*	*SPINK1*+AFP
Transcriptomics training dataset			
IDI	Reference	18.37% (14.04%–22.71%), *p* < 0.001	22.55% (19.16%–25.94%), *p* < 0.001
NRI	Reference	71.56% (60.16%–82.97%), *p* < 0.001	79.59% (69.04%–90.14%), *p* < 0.001
AIC	642.2	539.1	509.5
BIC	650.8	547.7	522.4
Transcriptomics validation dataset no. 1			
IDI	Reference	34.23% (28.99%–39.46%), *p* < 0.001	35.88% (31.17%–40.59%), *p* < 0.001
NRI	Reference	114.5% (98.56%–130.44%), *p* < 0.001	120.58% (105.9%–136.7%), *p* < 0.001
AIC	507.7825	347.85	340.42
BIC	515.74	355.8	352.36
Proteomics training dataset			
IDI	Reference	15.15% (10.38%–19.92%), *p* < 0.001	15.29% (10.56%–20.01%), *p* < 0.001
NRI	Reference	50.12% (34.16%–66.07%), *p* < 0.001	48.20% (31.81%–64.90%), *p* < 0.001
AIC	338.0	306.7	298.16
BIC	345.2	313.93	308.8

Comparison of the discrimination and goodness of fit of three predictive models for HCC in the different datasets.

IDI, integrated discrimination improvement; NRI, net reclassification improvement; AIC, Akaike information criterion; BIC, Bayesian information criterion

Since calibration reflects the extent to which a model correctly assesses the absolute incident or risk (35), we calculated the AIC and BIC, which revealed the following: AIC = *SPINK1* (539.1):AFP+*SPINK1* (509.5):AFP (642.2) and BIC = 547.7:522.4:650.8. These results indicate better calibration in the *SPINK1*-related model compared to that in AFP (**Table 1**). The calibration curves between the predicted and observed values for the *SPINK1*-associated models generally fitted well with the predicted probability of 0.3–1.0 along the *x*-axis compared to the AFP model alone, in which the goodness of fit between the two curves remained better only in the range 0.7–1.0 and exhibited poor calibration particularly within the range of <0.35, where a relatively low incidence rate of predicted HCC occurred, suggesting the poor sensitivity of AFP (**Figures 2B–D**). To evaluate clinical significance of these markers, we performed DCA, which revealed that the *SPINK1*-related models both had remarkably higher net clinical benefits within the high risk threshold probability range 0.1–0.8 relative to that of AFP (**Figure 2E**). Moreover, the DCA-derived CICs supported the aforementioned findings (**Figures 2F–H**).

**Figure 3:**
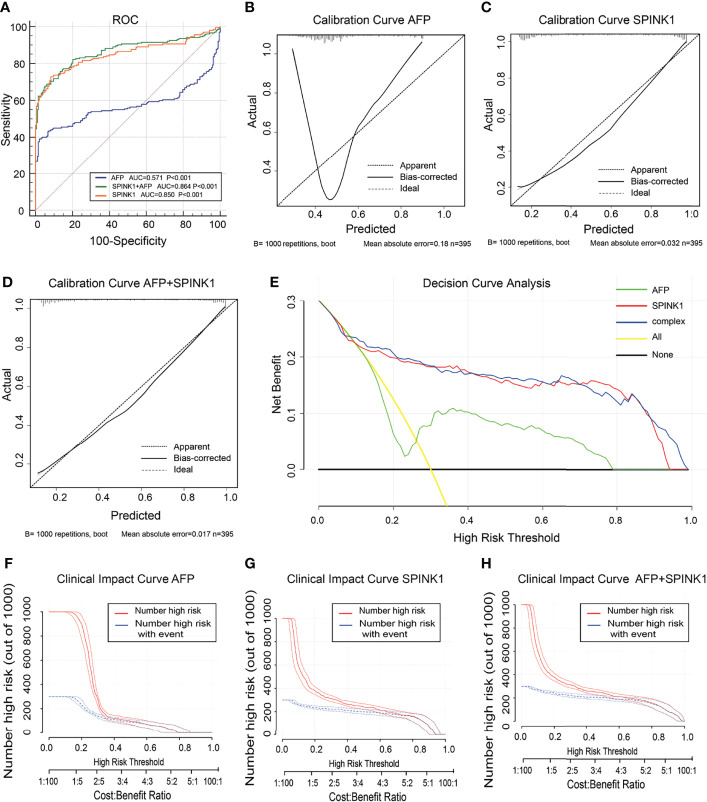
Discrimination and calibration of hepatocellular carcinoma (HCC) prediction models through transcriptomics analyses in the validation dataset no. 1. **(A)** Receiver operating characteristic (ROC) curves and area under the curve (AUC) in the *SPINK1*, *SPINK1* plus alpha-fetoprotein (AFP), and AFP models. **(B–D)** Calibration curves of AFP **(B)**, *SPINK1* **(C)**, and *SPINK1*+AFP **(D)**. **(E)** Clinical net benefits shown by decision curve analysis (DCA) of *SPINK1*, *SPINK1*+AFP, and AFP. **(F–H)** Comparison of the clinical benefits obtained from the clinical impact curve (CIC) analysis of each detection model.

In the analysis of the validation dataset, we chose two new, non-mixed available GEO and RNA-seq datasets that had been created by two specific groups and platforms for primary analysis (15). In the first validation dataset, the analyses led to significantly consistent results with the findings from the training dataset (**Figure 3**), as the discrimination capability of the *SPINK1*-related curves was as good as that shown in the discovery dataset, as well as the quality of calibration in the same setting. The analyzed data were described in the following order: *SPINK1* vs. *SPINK1*+AFP vs. AFP [AUC = 0.85:0.86:057, specificity = 91.71%:94.82%:97.93%, sensitivity = 72.77%:67.82%:39.11% (**Figure3A**); IDI = 1.34:1.36:reference, NRI = 214%:220%:reference) (**Table 1**)]. The calibration curves generally coincided with a much greater extent of the risk threshold probability at 0.2–1 in the *SPINK1*-related prediction models relative to that of AFP, which showed poor goodness of fit when the predicted risk probability was <0.3 (**Figures 3B–D**). In support of this, decreased values of AIC and BIC were calculated in these two models: AIC = 347.8:340.4:507.7 and BIC = 355.8:352.3:511.7 (**Table 1**). DCA and the CIC analysis also added more support to the two *SPINK1* patterns (**Figures 3E–H**). In the second validation dataset, the analyses generally showed consistent results with those derived from the training dataset (**Supplementary Figure S5**,**Table S2**).

### Proteomic Abundance of *SPINK1* in the Detection of HCC

**Figure 4:**
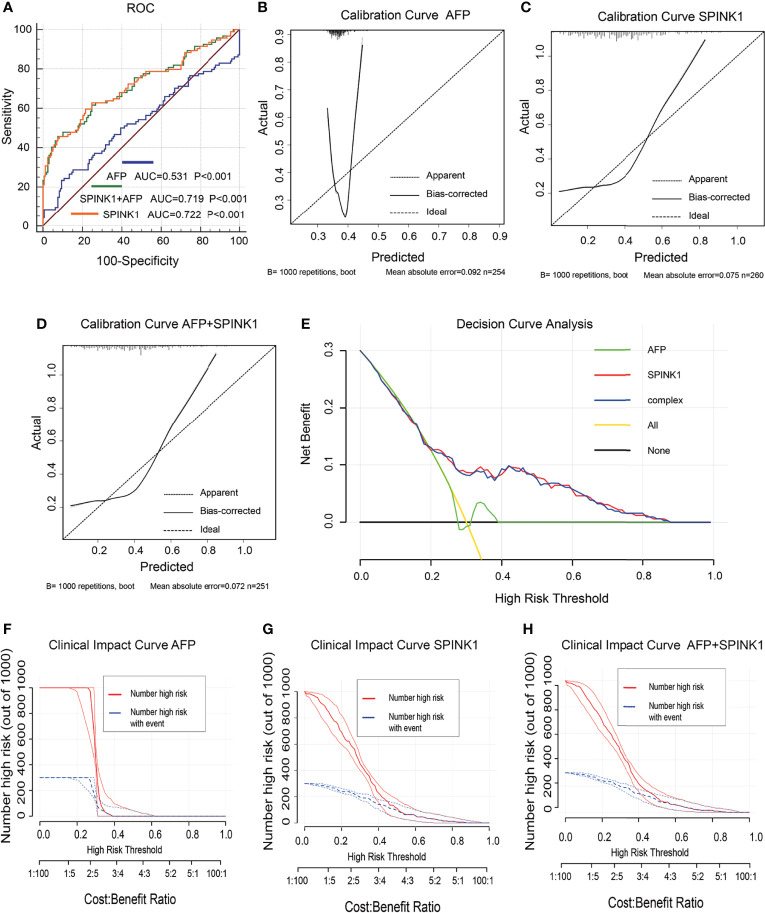
Discrimination and calibration of hepatocellular carcinoma (HCC) prediction models *via* proteomics analyses in the training dataset. **(A)** Receiver operating characteristic (ROC) curves and area under the curve (AUC) in the *SPINK1*, *SPINK1* plus alpha-fetoprotein (AFP), and AFP models. **(B–D)** Calibration curves of AFP **(B)**, *SPINK1* **(C)**, and *SPINK1*+AFP **(D)**. **(E)** Comparison of the decision curve analysis (DCA) of *SPINK1*, *SPINK1*+AFP, and AFP in terms of clinical net benefits. **(F–H)** Clinical benefits obtained from the clinical impact curve (CIC) analysis of each detection model.

In the training dataset, the proteomics analyses revealed similar trends to those shown in the previously described transcriptomics data. The *SPINK1* and *SPINK1*+AFP models exhibited a higher discriminatory efficacy than did AFP alone in stage I and II tumor samples: AUC = 0.72:0.72:0.53, specificity = 92.0%:92.4%:90.6%, sensitivity = 46.9%:45.7%:23.4% (**Figure 4A**). *SPINK1* rendered an almost equal specificity and a considerably elevated sensitivity relative to the AFP control, although the sensitivity values decreased for each of the three predictive patterns. The IDI results also strengthened the above findings by showing almost the same trend in this dataset, i.e., increased by 15.15% and 15.29% (IDI = 115.15%:115.29%:reference), while the NRI values showed increases of 50.12% and 48.20% compared to the AFP reference (NRI = 150.12%:148.20%:reference) (**Table 1**). The calibration of the predicted and observed values was remarkably improved with the inclusion of *SPINK1* compared to that of AFP alone, which displayed a poorly calibrated pattern (**Figures 4B–D**). Similarly, the decreased AIC and BIC values were observed correspondingly for the *SPINK1*-associated patterns (**Table 1**), reconfirming the better goodness of fit in these two *SPINK1* models (AIC = 306.7:298.16:338.0, BIC = 313.93:308.8:345.2). DCA also added more positive data to the *SPINK1*-related groups, indicating more clinical benefits obtained with the inclusion of *SPINK1* within the risk threshold probability of 0.2–0.9 (**Figure 4E**). The CICs clearly showed greatly improved clinical benefits in the abovementioned two settings within the range of predicted probability of 0.1–0.2, in which the incidence of HCC is quite low (**Figures 4F–H**). The protein level of *SPINK1* was also not correlated with that of AFP, indicating *SPINK1* as an independent predictive factor (*R* = −0.001; **Supplementary Figure S6**).

Although the transcriptomics and proteomics analyses of the expression patterns of *SPINK1* in HCC tissues laid the foundation for its qualification as a promising biomarker for the early detection of the disease, there probably are other hidden properties of *SPINK1* that greatly supported or contributed to its role in this scenario. Therefore, we next studied, using bench-scale experiments, some but not all of the tumorigenic phenotypes of *SPINK1* that were associated with the proliferation, clonal formation, migration, metastasis, chemoresistance, and anti-apoptosis of HCC cells. The following showed that the accumulated data not only favorably supported the function of *SPINK1* in the detection of HCC but also gradually led to the temporary conclusion that it may also be a possible molecular target in HCC therapies based on some of its unique biological features of cancer.

### Stimulation of Cellular Proliferation and Clonal Formation by *SPINK1*

**Figure 5:**
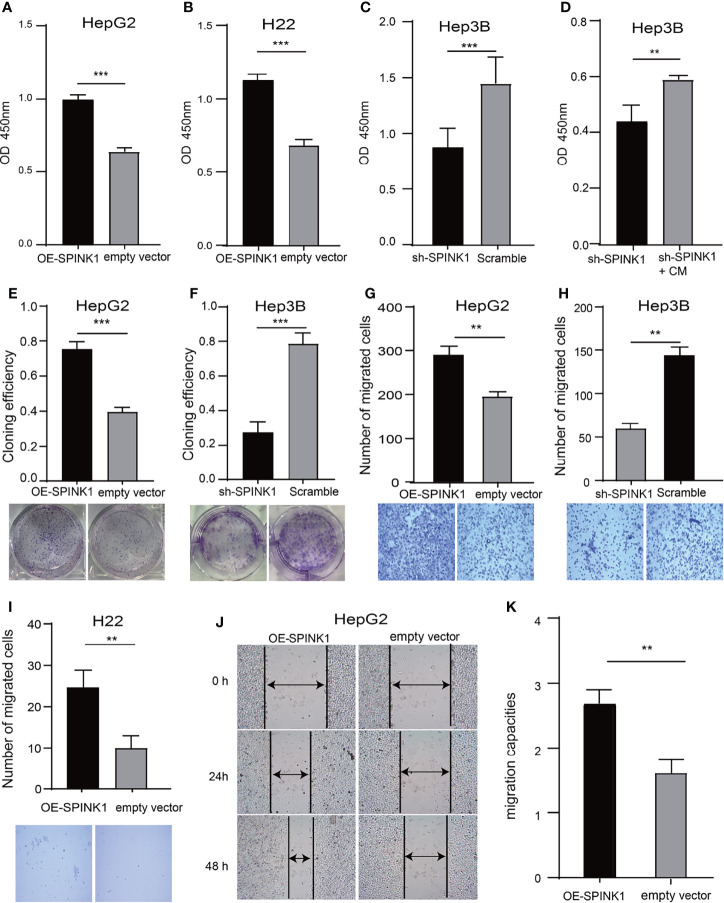
*SPINK1*’s regulation on proliferation, clonogenic formation and migration of *SPINK1*-OE and *SPINK1*-silencing HCC cells. **(A, B)** CCK-8 assays for metabolic and proliferative activities in stable *SPINK1*-OE HepG2 and *SPINK1*-OE H22 cells. **(C, D)** Proliferative capability and corresponding phenotype rescue after addition of conditioned medium (CM) for *SPINK1*-silencing Hep3B cells. **(E, F)** Clonogenic formation in *SPINK1*-OE HepG2 and knockdown Hep3B HCC cells, along with images of formed clones following inoculation of corresponding *SPINK1*-OE HepG2, *SPINK1*-silencing H3B and control cells for two weeks. **(G–I)** Transwell assays for *SPINK1*-OE HepG2, *SPINK1*-OE H22 and *SPINK1* knockdown Hep3B cells, with photos that showed cells passing through well’s pores both in control and experimental groups, respectively. **(J, K)** Images and bar chart for measurement of migration distances of *SPINK1*-OE HepG2 cells 24 and 48 hrs post-scratching in Wound-healing assay (Scratch test). Error bars represent mean ± standard deviation of independent experiments or the experiments performed in triplicates either in 6-well or 96-well plates. OD, optical density; **, P < 0.01; ***, P < 0.001.

We determined the possible carcinogenic phenotypes of *SPINK1* that were possibly responsible for its predictive capability in HCC at the cellular level. We initially created the permanently *SPINK1*-OE HepG2 and H22 cell strains and silenced the *SPINK1* Hep3B cell line using the C2 shRNA vector, the most efficient *SPINK1* knockdown construct (**Supplementary Figure S7**). Compared with the control, the metabolic and proliferative capabilities increased significantly in *SPINK1*-OE HepG2 and H22 cells and decreased considerably in *SPINK1* knockdown Hep3B cells (*p* < 0.01; **Figures 5A–C**). The clonogenic assay showed that the number of colonies formed in *SPINK1*-OE HepG2 cells was greater than that in empty vector controls, whereas *SPINK1* knockdown Hep3B cells rendered a significant inhibitory effect on the number of cell colonies formed (*p* < 0.01; **Figures 5E, F**).

### Rescue of shRNA-Mediated Phenotypes by Conditioned Medium

To further confirm the indispensable role of *SPINK1* in the growth of HCC cells, we performed the “rescue” experiment in *SPINK1* shRNA-transfected Hep3B cells. It was found that the survival rate of Hep3B cells was significantly decreased following transfection of the *SPINK1* shRNA lentiviral construct (**Figure 5C**). However, the CCK-8 assay showed that the addition of exogenous *SPINK1* CM could remarkably rescue Hep3B cells from the proliferation-inhibitory state, while the vector CM was unable to reach this outcome (*p* < 0.01; **Figure 5D**), suggesting the specificity of *SPINK1* as a survival factor in the growth of Hep3B cells.

### Migration and Invasion *via SPINK1* Regulation

The results of the Transwell assays demonstrated that the number of migrated cells *via* the pores in the *SPINK1*-OE HepG2 and H22 groups were much more than those in the respective controls, whereas fewer number of migrated cells could be observed in *SPINK1* knockdown Hep3B cells (*p* < 0.01; **Figures 5G–I**). In addition, the scratch test also showed similar results (*p* < 0.01; **Figures 5J, K**). Together, these data suggest that *SPINK1* possesses the ability to promote the proliferation, clonal formation, migration, and invasion of HCC cells.

### *SPINK1*-Associated Tumorigenesis and Metastasis *In Vivo*

**Figure 6:**
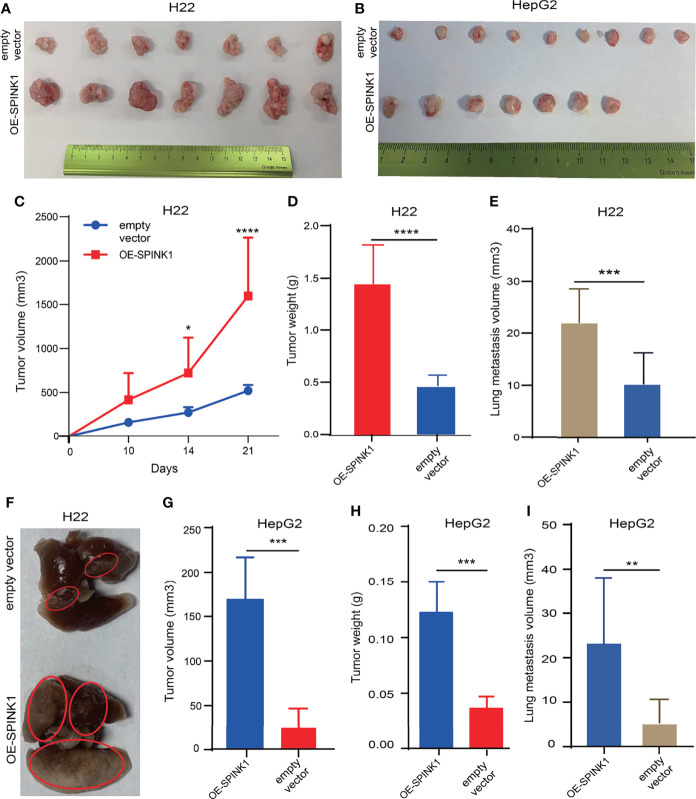
*SPINK1*-stimulated tumorigenesis , metastasis in vivo experiments. **(A, B)** Photos for homografts and xenografts generated from subcutaneous injection of murine *SPINK1*-OE H22 and human *SPINK1*-OE HepG2 and corresponding control cells in flank region of normal immuno-competent Balb/c mice ( H22: n1control = 7, n2 test = 7; HepG2: n1 control = 9, n2 test = 7). **(C, D)** Alterations of tumor volume and weight during growth period in Balb/c mice of ‘*SPINK1*-OE H22’ and control groups. **(E, F)** Estimated volume and images of metastatic lung nodules at 4 weeks post-injection of *SPINK1*-OE H22 and respective control H22 cells via murine tail vein. **(G, H)** Tumor volume and weight measurement in the process of growth in Balb/c mice (*SPINK1*- OE HepG2 cells). **(I)** Measurement of lung metastasis via estimated volume of pulmonary tumor nodules (*SPINK1*-OE HepG2 and control HepG2 cells). Error bars represent mean ± standard deviation of one independent experiment. *, P < 0.05; **, P < 0.01; ***, P < 0.001; ****, P < 0.0001.

We attempted to reproduce some of the aforementioned *in vitro* observation in *in vivo* experiments. To simulate the tumorigenic and metastatic capacities of *SPINK1* under immunocompetent circumstances, we employed murine HCC models of implanted homograft and metastasis using *SPINK1*-OE H22 cells. A significant increment in the tumor size, on average, was observed weekly, and this trend gradually reached the peak at 3 weeks after implantation of *SPINK1*-OE H22 tumor cells in mice (**Figure 6A**). The tumor graft volume and the weight of *SPINK1*-OE H22 mice were significantly higher than those of the control (*p* < 0.01; **Figures 6C, D**). Moreover, we estimated the total volumes of the metastatic lung nodules; the results showed greater numbers in the *SPINK1*-OE cell-injected mice compared to the H22 control animals 4 weeks after tail vein administration of *SPINK1*-OE H22 cells, suggesting the pro-metastatic capability of *SPINK1* (*p* < 0.01; **Figures 6E, F**). Simulating the real immunocompetent environment, we also injected human *SPINK1*-OE HepG2 cells subcutaneously in the flank of Balb/c mice. Despite xenograft rejection, *SPINK1* overexpression also promoted the tumorigenesis and metastasis of HepG2 cells in mice under normal immunological conditions (*p* < 0.01; **Figures 6B, G, H, I**).

### *SPINK1* Delivery of Chemoresistance and Anti-Apoptosis

**Figure 7:**
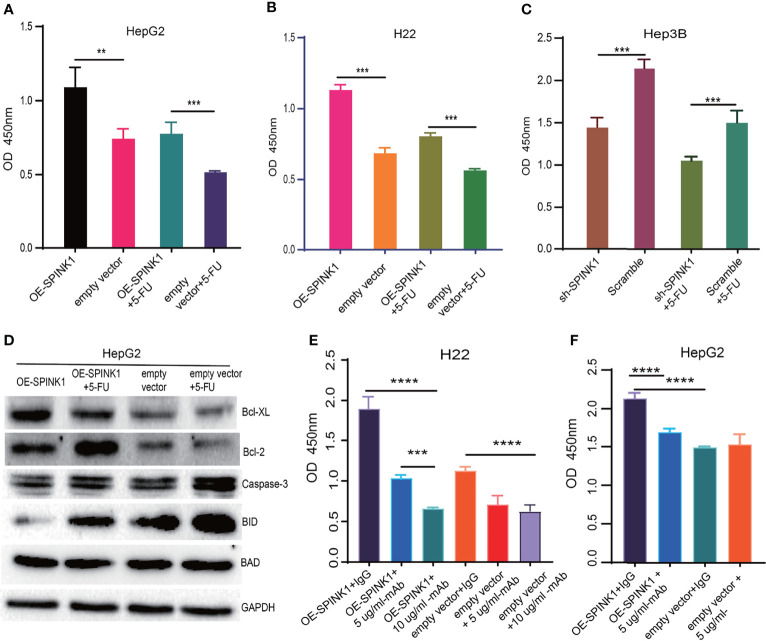
*SPINK1*-regulated chemoresistance, anti-apoptosis and *SPINK1*-mAb targeting experiments. **(A, B)** *SPINK1* enhanced chemoresistance in stable *SPINK1*-OE HepG2 and *SPINK1*-OE H22 cells following 24 h of exposure to 5-FU; **(C)** Decreased chemoresistance to 5-FU in *SPINK1* knockdown Hep3B cells 24 h post-5-FU treatment. **(D)** Examination of apoptosis-related molecules including main pro- (Caspase 3, Bid and Bad) and anti-apoptotic regulators (Bcl-XL and Bcl-2) using Western blot in *SPINK1*-OE HepG2 and empty-vector control cells with or without 5-FU addition. **(E, F)** Targeting *SPINK1* using anti-human *SPINK1*- mAb in *SPINK1*- OE H22 and - OE HepG2 cells, with IgG as an isotype control. Error bars represent mean ± standard deviation of independent experiments or the experiments performed in triplicates either in 6-well or 96-well plates. OD, optical density; **, P < 0.01; ***, P < 0.001; ****, P < 0.0001.

Closely associated with the nature of the tumor, chemoresistance has been reported to be induced in the process of chemotherapy for HCC treatment. As previous data demonstrated the pro-survival feature of *SPINK1*, we then wondered whether *SPINK1* confers resistance to chemotherapeutic drugs in HCC cells. We treated both *SPINK1*-OE HepG2 and H22 cells and *SPINK1*-silencing Hep3B cells with or without 2.5 µg/ml 5-fluorouracil (5-FU), an accepted dose used in *in vitro* chemoresistance-related experiments. The CCK-8 assay showed a significant increase in the number of *SPINK1*-OE HepG2 and H22 cells with 5-FU treatment relative to the vector control cells, whereas a remarkable decrement could be detected in *SPINK1*-silencing Hep3B cells treated with 5-FU (*p* < 0.01; **Figures 7A–C**) compared to the scramble control. Thus, despite its unknown mechanisms, *SPINK1* was able to increase chemoresistance in *SPINK1*-OE HCC cells *in vitro*.

To investigate the possible mechanisms through which *SPINK1* promoted the growth of HCC and conferred chemoresistance with 5-FU administration, we examined the apoptotic status of *SPINK1*-OE HepG2 cells with or without 5-FU treatment. A number of apoptotic players, including pro- and anti-apoptotic molecular signals, were analyzed: caspase-3, Bid, Bad, Bcl-2, and Bcl-XL. Interestingly, the anti-apoptotic levels of Bcl-2 and BcL-XL were significantly upregulated in *SPINK1*-OE and 5-FU-administered HepG2 OE cells compared with the empty vector HepG2 controls, whereas the pro-apoptotic molecule Bid was remarkably downregulated in these cells (**Figure 7D**). The expression level of caspase-3, one of the key factors inducing apoptosis, remained decreased in *SPINK1*-OE HepG2 cells treated with or without 5-FU (**Figure 7D**). All of these results suggest that the imbalance between the anti-apoptotic and pro-apoptotic power may possibly favor the former “anti-aspect” (**Figure 7D**) in respective circumstances. Together, the *SPINK1*-associated inhibition of apoptosis may be one of the explanations for the *SPINK1*-induced overgrowth and chemoresistance in HCC cells.

### Targeting *SPINK1* in HCC Cells

Analyses of the acquired experimental data provided more understanding on *SPINK1*. We therefore further explored the likelihood of *SPINK1* as a target molecule in targeted therapeutics for HCC, as we verified its targeting exhibited during HCC progression and development. The efficacy of HCC targeted therapy currently remains quite limited primarily due to the difficulty in finding a “precision” molecular target (5). To prove the possibility of *SPINK1* as an actionable target, we performed *in vitro SPINK1*-targeting experiment using anti-human *SPINK1* mAb. As expected, the *SPINK1* mAb significantly (5 µg/ml) decreased the proliferation of *SPINK1*-OE HepG2 and H22 cells by 25% and 50%, respectively, as well as caused a proliferative decrement of 40% in H22 empty vector control cells, compared to a control monoclonal IgG antibody (*p* < 0.01; **Figures 7E**, **F**), suggesting considerable efficacy at targeting *SPINK1*. Interestingly, the increased concentration of the *SPINK1* antibody (10 µg/ml) could even significantly inhibit the growth rate by 75% in *SPINK1*-OE H22 and by 45% in H22 control cells (**Figure 7E**). These data may indicate the potential of *SPINK1* as a promising target molecule in the management of HCC.

### Exploration of the Underlying Molecular Signaling Pathways

**Figure 8:**
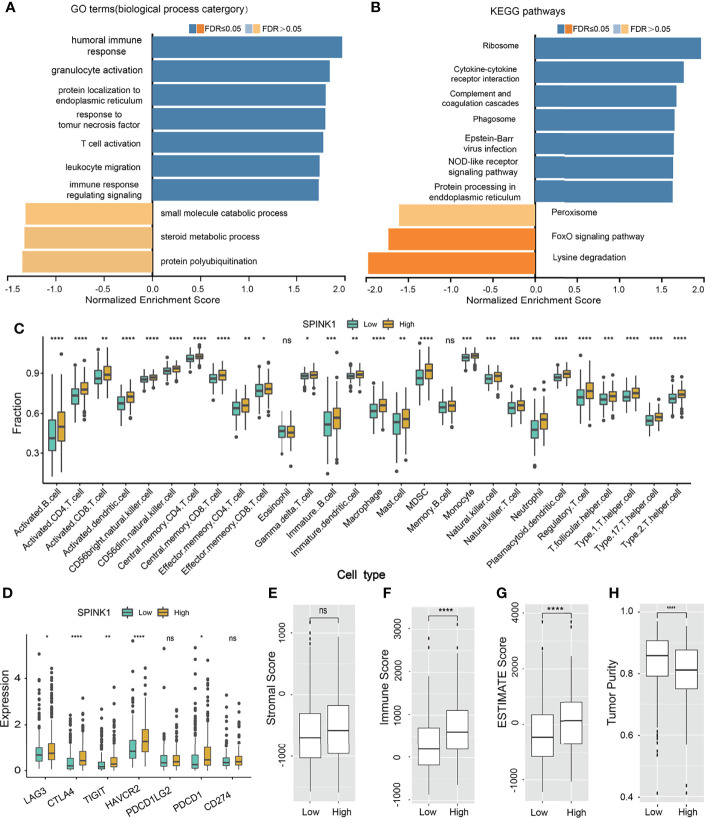
*SPINK1*-related gene set enrichment analyses (GSEA) and *SPINK1*, tumor infiltrating microenvironment (TIME) **(A, B)** *SPINK1*-associated gene set enrichment analyses (GSEA) of GO terms (biological process categories) and KEGG pathways using HCC RNAseq data from LinkedOmics database. **(C)** Relationships among *SPINK1* expression abundance (low-blue vs high-yellow expression groups) and those of 28 subtypes of tumor infiltrating immune cells in terms of compositions and contents in tissue specimens of HCC patients from TCGA dataset. **(D)**Correlations among *SPINK1* and other seven immune checkpoints molecu1es in *SPINK1*- low and -high abundance groups of above-mentioned HCC samples. **(E–H)** Correlations among *SPINK1* and scores of ‘stromal’, ‘immune’, ‘estimate’ and ‘tumor purity’ in *SPINK1*- low and -high expression groups of same cohort of patient samples, respectively. NS, no significance; *, P < 0.05; **, P < 0.01; ***, P < 0.001; ****, P < 0.0001.

We wanted to discover the underlying molecular mechanisms that contributed to the tumorigenic features and targetable characteristics of *SPINK1* in the *in vitro* and *in vivo* experiments. For this purpose, we used the LinkedOmics online tool to determine the co-expression genes that positively or negatively correlated with *SPINK1* expression using the RNA-seq data of 371 HCC patients in the TCGA cohort (20). Subsequently, GSEA (GO and KEGG) was performed using this tool. The GO analysis revealed that the most *SPINK1*-related biological processes to the differentially expressed related genes were “T-cell activation,” “response to tumor necrosis factor,” “immune response regulating signaling pathway,” “humoral immune response,” and “leukocyte migration,” which are almost all required for delivering antitumor immunity (**Figure 8A**). On the other hand, KEGG signaling pathway analysis showed that these genes were mainly enriched in “cytokine–cytokine receptor interaction,” “complement cascade,” “phagosome,” and “NOD-like receptor signaling pathway,” which are also associated with the delivery of antitumor response (**Figure 8B**). Thus, both GO and KEGG analyses revealed the positive involvement of *SPINK1* in the regulation of an active immune response, which was likely aimed at directly or indirectly resisting HCC.

The data from the GO and KEGG analyses inspired the realization that *SPINK1* has been engaged in immune response-associated biological processes and signaling pathways in the development of HCC and that it might be a suitable biomarker for the prediction of a “hot” tumor immune microenvironment (TIME) and the response to ICB therapy for HCC. Currently, based on our literature search, there are no efficacious predictive biomarkers for ICB remedy in this regard (5). Thus, we decided to proceed to the last assumption of this investigation: test the possibility of *SPINK1* in the prediction of response to HCC immunotherapy.

### Correlation Between *SPINK1* and Infiltrating Immune Cells

To probe the relationship between *SPINK1* and the tumor-infiltrating immune cells in the HCC specimens from the TCGA dataset, which was currently found to be critical in predicting the effectiveness of ICB therapy (36, 37), we analyzed 28 types of infiltrating immune cells within the HCC samples using RNA-seq data. Thereafter, we examined the correlation between the expression of *SPINK1* and the intrinsically infiltrating immune cell subtypes. The statistical analyses showed the relative proportions of the 28 types of immune cells in each of the HCC samples (**Supplementary Figure S8**). The dominantly infiltrating immune cells were activated CD4 T, CD8 T natural killer, effector memory CD8 T, dendritic, and type 1 and 2 T helper cells (**Supplementary Figure S8**). All these subtypes of immune cells play essential roles in the antigen-specific response to tumor immunity in the host (37). Interestingly, the transcriptomic expression of *SPINK1* was positively correlated with most of the infiltrating immune cells, particularly activated CD4 T, CD8 T, effector memory CD4 T, effector CD8 T, and activated dendritic cells (**Figure 8C**). These immune cells are believed to be involved in CD8^+^ T-cell-mediated antitumor immune response (36). Collectively, *SPINK1* abundance was positively associated with most of the important subtypes of infiltrating immune cells.

### Associations Among *SPINK1*, Immune Checkpoints, and Tumor Mutation Burden

We also tested the likelihood *SPINK1* being a predictor of the efficacy of ICB therapy, a burgeoning field in HCC treatment (10). We examined the correlation between the transcriptomic expression of *SPINK1* and a number of critical ICPs—PD-1, LAG-3, TIM-3, TIGIT, and CTLA-4—which are the likely signatures for the response to ICB in cancer immunotherapy (21, 38). Unexpectedly, a high level of *SPINK1* expression was found to be significantly and positively correlated with the high expressions of PD-1, LAG-3, TIM-3, TIGIT, and CTLA-4 in the tumor samples from the TCGA cohort (**Figure 8D**). The expression of PD-L1, a recommended potential predictor for immunotherapy vulnerability (39), was not positively associated with that of *SPINK1* in the TCGA cohort (**Figure 8D**). We then analyzed the association between *SPINK1* and TMB, a temporarily used marker for the prediction of ICB efficacy. The results showed no correlation between them (**Supplementary Figure S9**). Altogether, investigation of the RNA-seq data of the HCC samples from the TCGA database revealed a positive correlation between *SPINK1* and the expression of some of the important immune checkpoint molecules, suggesting the necessity of reprogramming the immunosuppressive milieu of this category of patients for better prognosis (21).

### Patterns of Connection Between *SPINK1* and the ESTIMATE and Immune Cell Scores

To further evaluate the association between *SPINK1* and TIME, laying the possible foundation to predict the efficacy of ICB therapy in HCC patients, we analyzed a few important parameters from different angles using ESTIMATE software: the “immune score” (proportion of immune cells), “stromal score” (proportion of stromal cells), “ESTIMATE score” (proportions of immune cells plus stromal cells), and “tumor purity” (proportions of tumor cells/the total) of the tumor specimens (40) (**Figures 8E–H**). Except for the expression of *SPINK1* not being associated with the stromal score (non-immune cells) and being negatively correlated with tumor purity, the ESTIMATE and immune scores were positively correlated with the abundance of *SPINK1* (**Figures 8E–H**), suggesting that *SPINK1* could be considered as an indicator of the unique reflection of a hot or immune cell-infiltrating tumor microenvironment (41), with the likelihood of a positive response to ICB in HCC patients (42).

## Discussion

Beginning with mining an optimal biomarker for the early detection of HCC in this study, we then discovered some of the “targetable” characteristics of *SPINK1* through phenotypic analyses of its tumorigenic biological characteristics before finally determining that *SPINK1* is also eligible for predicting the response to ICB treatment. The three *SPINK1*-associated research issues were resolved through the analyses of data derived from genomics, transcriptomics, and proteomics datasets and from real bench-scale experiments.

There have been few reports, if any, on the integration of multi-omics and phenotypic analyses of *SPINK1* for the detection of early-stage (I and II) HCC, particularly compared to AFP, a routinely used marker for HCC (43, 44). Holah et al. and Zhu et al. found that the protein expression levels of *SPINK1* in sera were remarkably higher in patients with liver cirrhosis and HCC (stages I–IV) than in those with chronic hepatitis (45, 46). With the small number of patient specimens, however, the previously reported data showed no or very limited discriminatory accuracy and calibrated analyses of *SPINK1* in the early diagnosis of the disease (44–46).

We used ROC plots and showed that the *SPINK1* transcripts displayed a much better discriminatory capability than AFP for early (stages I and II) diagnosis at the transcriptomic level (47). These findings indirectly re-confirmed previous reports of the low sensitivity of serum AFP as a diagnostic criterion for HCC (2–4, 33, 34). Impressively, the sensitivity increased greatly when AFP was replaced by *SPINK1* or when *SPINK1* was combined with AFP in the prediction model. Moreover, the AUC, specificity, and sensitivity of the *SPINK1*-related models for stage I and II HCC were better than those for stage I–IV patients of the same cohorts, implying the superiority of *SPINK1* in the early diagnosis of HCC (stages I and II) than that in the late stages (III and IV).

It is worth noting that the calibration curves of the *SPINK1*-related models in the analysis of the first validation dataset were well fitted, indicating the generality of *SPINK1* as a better diagnostic biomarker for HCC. The results of the Hosmer–Lemeshow test in the second validation dataset showed an increased *p*-value (>0.05), which indicated an overall better fit of the *SPINK1*-related models to the observations.

Our corresponding findings for *SPINK1* were contrary to those of a previous report by Yan and Chen describing *SPINK1* overexpression as only correlated with late-stage HCC (stages IIIB to IV) (24), possibly due to the small number of samples included in their study. Some researchers have also stated that the addition of one or more serum biomarkers to AFP, such as des-gamma-carboxy prothrombin (DCP), glypican-3 (GPC3), and Golgi protein 73 (GP73), does not significantly improve its diagnostic accuracy for HCC (48). In contrast, our data clearly demonstrated that *SPINK1* and the combination of *SPINK1* and AFP both showed more beneficial outcomes than AFP alone using the tissue samples, implying the possible advantages of using *SPINK1* over the other currently recommended biomarkers for early detection (48).

For the first time, to our knowledge, proteomics was integrated with transcriptomics datasets for the analysis of the early detection of HCC using *SPINK1*. It was noteworthy that the discrimination curve of AFP was poor, possibly due to the selection of early-stage (I and II) tumor samples, for which the detection using AFP was not sensitive enough. AFP also exhibited poor calibration on the prediction curve in the proteomics dataset. Although the discriminatory ability of the *SPINK1*-associated models was much better than that of AFP, it was not as good as that from the transcriptomics study. One of the reasons for this phenomenon might be related to the limited number of samples from early-stage patients. Nevertheless, all the other indices including AIC, BIC, IDI, and NRI still showed the same positive trends as those of transcriptomics in the proteomics analyses.

In fact, the switch from “early detection” to “targeted therapy” naturally occurred out of the continuously obtained data during further phenotype analyses of the tumorigenic characteristics of *SPINK1*. We did not find reports on the targeting of *SPINK1* in HCC within our PubMed search, although Ateeq et al. experimentally showed that the human anti-*SPINK1* antibody against prostate cancer could reduce tumor growth (49). Currently, the efficacy of targeted therapy for HCC remains quite limited primarily due to the difficulty in finding a “precision” molecular target (5). Our *in vitro* and *in vivo* experimental data convincingly demonstrated the role of *SPINK1* in tumor growth, invasion, chemoresistance, anti-apoptosis, and metastasis and the favorable effect of the anti-*SPINK1* antibody on the anti-proliferation of HCC cells. These findings will likely lay the foundation for providing a new targetable molecule in order to improve the efficacy of targeted treatment for the management of HCC.

The *SPINK1*-related immune response biological processes and signaling routes, including T-cell activation based on GO and KEGG analyses, led us to focus on the next ICB-associated concern. Currently, the biomarkers for the identification of ICB response remain critically important and challenging in the management of HCC (50). To our knowledge, for the first time, we have shown *SPINK1* as a potentially practicable and accessible biomarker that could be utilized for the prediction of the presence of a hot immunogenic environment (40) in HCC tissues and may likely be useful for starting ICB treatment. Our data clearly showed that the high abundance of *SPINK1* corresponded with the high levels of CD4^+^ and CD8^+^ effector T lymphocytes, as well as activated dendritic and natural killer cells in the same tumor samples. There has been a general consensus that tumor-infiltrating CD4^+^ and CD8^+^ effector T lymphocytes are mediators of the response to ICB and that these cells are primed through tumor antigen presentation by dendritic cells, while natural killer cells play important roles in the immune surveillance of tumors (10). Specifically, the overexpression of *SPINK1* in the samples suggested the possibility of a hot tumor, in which effector T cells may play the central part in the antitumor response; therefore, patients with a hot TIME are more likely to respond to immunotherapy (40). Interestingly, the high abundance of *SPINK1* was also exclusively associated with the high levels of ICPs in our analyses, and these ICPs were thought to be involved in the mechanisms by which cancer cells disguise themselves as regular components of the human body (51). The assumption was that inhibitors against ICPs can reinvigorate anticancer immunity, which are currently the standard of care in a number of malignancies (52). Thus, preexisting abundant ICPs are important signals for the delivery of ICB therapy (53). Moreover, GSEA (GO) revealed that *SPINK1* participated primarily in the biological process of antitumor-related immune response, including response to T-cell activation, and humoral immune response, which was associated with favorable outcomes when individuals undergo immunotherapy (54). Thus, the high expression of *SPINK1* may be an accurate parameter to indicate the appropriate time point for ICB intervention.

Notably, there was no positive correlation between *SPINK1* and PD-L1 in our study. Havel et al. reported that a number of lung cancer patients with PD-L1-negative tumors still responded to ICB and that, in some tumor types, the expression of PD-L1 did not correlate with response to treatment (55). El-Khoueiry et al. also showed that the tumoral expression of PD-L1 was not predictive of the response to nivolumab or pembrolizumab in HCC treatment (56). Whether PD-L1 could be considered as a marker for the prediction of ICB vulnerability is a topic for further investigation in HCC management.

In our analyses, the group with a high expression of *SPINK1* did not reveal a positive correlation with TMB, which may increase the likelihood of ICB response, as described by other researchers in cancer control (55). This discrepancy was possibly due to the general low TMB in HCC and the unsuitability of TMB as a biomarker to predict ICB response in this regard (57).

The data on the immune, stromal, and ESTIMATE scores and tumor purity were generally consistent with previous results, indicating that the high abundance of *SPINK1* suggests a “hot” state of accumulation of highly infiltrating immune cells, termed a “better” immune score. Although ICB therapeutics including ipilumimab and nivolumab alter the T-lymphocyte checkpoint control and may be particularly efficacious in tumors with intrinsically high levels of infiltrating leukocytes, further subclassification of the lineage features of infiltrating cells, such as discriminating between various leukocytes, may possibly represent a more actionable characteristic of clinical application (22). Specifically, more data are still needed to determine whether the ESTIMATE immune score is a more appropriate parameter for the prediction of ICB response.

Some limitations in the analyses of this investigation should be noted. Firstly, we lacked clinical data with respect to the confirmed significance of the level of *SPINK1* in serum for the early detection of patients with HCC, which is the most accessible and practical test in the laboratory for cancer assessment. Secondly, the molecular targeting experiment using the anti-human *SPINK1* antibody was performed only at the cellular level *in vitro* rather than in an *in vivo* rodent model. Thirdly, the preliminary data derived from the GO and KEGG analyses deciphered in part why *SPINK1* could be a potential biomarker for the prediction of an immunologically hot tumor, which is possibly suitable for subsequent ICB therapy response in HCC (40). However, there were no controlled clinical data regarding the association between transcriptomic *SPINK1* and the response outcomes of patients with HCC during the ICB treatment. Further experimental and clinical evidence needs to be accumulated in order to reach definite conclusions. Fourthly, all of the transcriptomics and proteomics data were collected from varied batches of patients with HCC who may even be from the same or different hospitals, meaning that we were not able to avoid selection bias and imbalances in this study, which may have resulted in the precision of the obtained results to some extent (58).

Through a number of dry and wet experiments, we have demonstrated that *SPINK1* could act as a potential biomarker for the early detection, targeted therapeutics, and the prediction of ICB response in the management of HCC. Moreover, the combination of targeted therapeutics and ICB immunotherapy has become the forefront in HCC management (5). Thus, aside from being a “target,” there is a rational expectation to consider *SPINK1*-oriented ICB plus *SPINK1*-targeting therapeutics in the future. However, this temporary conclusion requires prospective validation instead of using tumor tissue specimens, a routinely accessible serum marker used in large-scale clinical trials, which should be applied in this regard before reaching definite conclusions. Hopefully, the application of *SPINK1* would pave a new avenue for the diagnosis and treatment of HCC.

## Data Availability Statement

The datasets presented in this study can be found in online repositories. The names of the repository/repositories and accession number(s) can be found in the article/**Supplementary Material**.

## Ethics Statement

The animal study was reviewed and approved by Animal Care and Use Committee of Dalian Medical University.

## Author Contributions

JJ and JW: conception and design and manuscript writing. JJ and LG: performed the experiments. JJ: collection and assembly of data. JJ, LG, YL, ZY, YW, XG, RM, RL, TL, ZT, and JW: data analysis and interpretation. All authors are accountable for all aspects of the work. All authors contributed to the article and approved the submitted version.

## Funding

This study was partially sponsored by Dalian Medical University.

## Conflict of Interest

The authors declare that the research was conducted in the absence of any commercial or financial relationships that could be construed as a potential conflict of interest.

## Publisher’s Note

All claims expressed in this article are solely those of the authors and do not necessarily represent those of their affiliated organizations, or those of the publisher, the editors and the reviewers. Any product that may be evaluated in this article, or claim that may be made by its manufacturer, is not guaranteed or endorsed by the publisher.
