# Supplementary material for: Serine Protease Inhibitor Kazal Type 1, A Potential Biomarker for the Early Detection, Targeting, and Prediction of Response to Immune Checkpoint Blockade Therapies in Hepatocellular Carcinoma
Source: Front Immunol. 2022 Jul 18;13:923031. doi: 10.3389/fimmu.2022.923031 (PMC9341429; doi:10.3389/fimmu.2022.923031)

|  | | | |
| --- | --- | --- | --- |
| GSE Dataset | HCC | Normal liver | Total |
| GSE101685 | 8 | 24 | 32 |
| GSE102079 | 152 | 105 | 257 |
| GSE107170 | 83 | 0 | 83 |
| GSE14323 | 55 | 19 | 74 |
| GSE14520 | 225 | 220 | 445 |
| GSE22405 | 24 | 24 | 48 |
| GSE29721 | 10 | 10 | 20 |
| GSE31370 | 15 | 5 | 20 |
| GSE39791 | 72 | 72 | 144 |
| GSE41804 | 20 | 20 | 40 |
| GSE45267 | 48 | 39 | 87 |
| GSE46408 | 5 | 6 | 11 |
| GSE51401 | 30 | 34 | 64 |
| GSE54236 | 79 | 0 | 79 |
| GSE57957 | 39 | 39 | 78 |
| GSE62232 | 81 | 10 | 91 |
| GSE69715 | 37 | 0 | 37 |
| GSE76427 | 82 | 52 | 134 |
| GSE84402 | 14 | 14 | 28 |
| GSE84598 | 21 | 22 | 43 |
| GSE89377 | 40 | 13 | 53 |
| GSE98383 | 16 | 0 | 16 |
| Total | 1156 | 728 | 1884 |

**Supplementary Table.1. List of NCBI-GEO datasets used for transcriptomics analyses of stage I-IV HCC specimens including 1156 HCC and 728 normal liver tissue.**

**Supplementary Table.2**. Discrimination and calibration of three detective models

|  | AFP | SPINK1 | SPINK1+AFP |  |
| --- | --- | --- | --- | --- |
| **Transcriptomics analyses in a data set containing 1884 stage I-IV HCC samples** | | | |  |
| IDI | reference | 14.99% [13.19%-16.8%] P<0.001 | 16.46% [14.87%-18.05%] P<0.001 |  |
| NRI | reference | 66.18% [57.62%-74.75%] P<0.001 | 87.66% [79.46%-95.86%] P<0.001 |  |
| AIC | 2358.5 | 2058.8 | 2021.3 |  |
| BIC | 2369.5 | 2069.9 | 2037.9 |  |
| **Transcriptomics validation data set No.2** | | | | |
| IDI | reference | 27.97% [19.15%-36.8%] P<0.001 | 35.86% [28.89%-42.82%] P<0.001 | |
| NRI | reference | 71.9% [55.87%-87.92%] P<0.001 | 79.50% [65.38%-93.61%] P<0.001 | |
| AIC | 312.2 | 224.9 | 200.7 | |
| BIC | 319.4 | 232.2 | 211.5 | |

Comparison in discrimination and goodness of fit of three predictive models for HCC data set containing 1884 stage I-IV HCC samples and a transcriptomics validation data set No.2.

**Supplementary Fig.1.** SPINK1 cDNA expression in tumor and non-tumor tissues. (A-D), cDNA microarray analyses for SPINK1 expression in HCC tissue samples, compared with non-cancerous liver specimen in four cohorts of varied numbers of patients from Oncomine database. (E) Immunohistochemical staining of SPINK1 in HCC tissue section versus normal control. (F) cDNA Expression of SPINK1 based on tumor stage in LIHC (Liver hepatocellular carcinoma). **(**G)cDNA Expression of SPINK1 based on tumor grade in LIHC. *, *P* < 0.05; **, *P* < 0.01; ***, *P* < 0.001; ****, *P* < 0.0001.


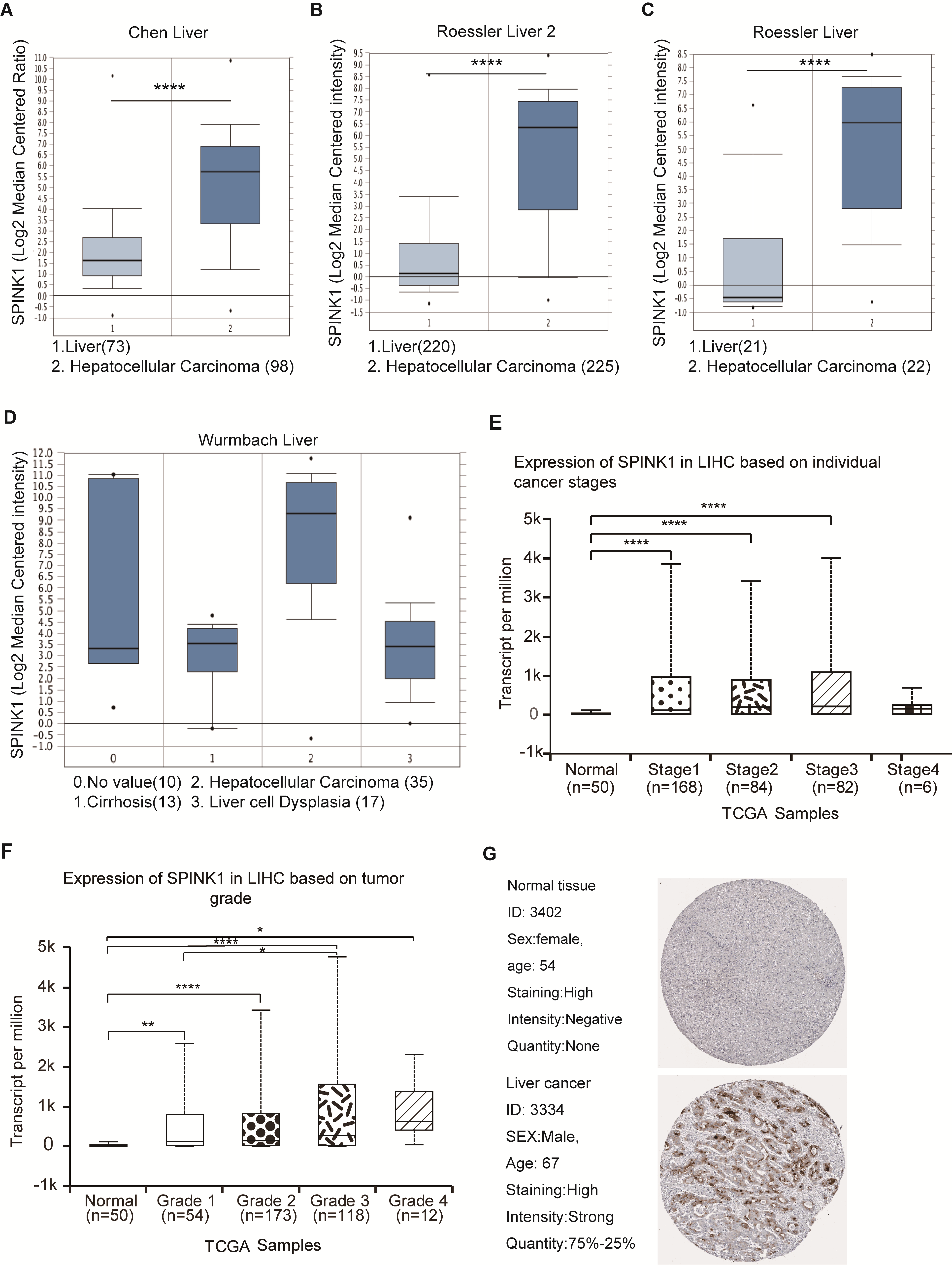


**Supplementary Fig.2.** Top (1-25) over-expressed genes in the tissues samples of liver hepatocellular carcinoma.


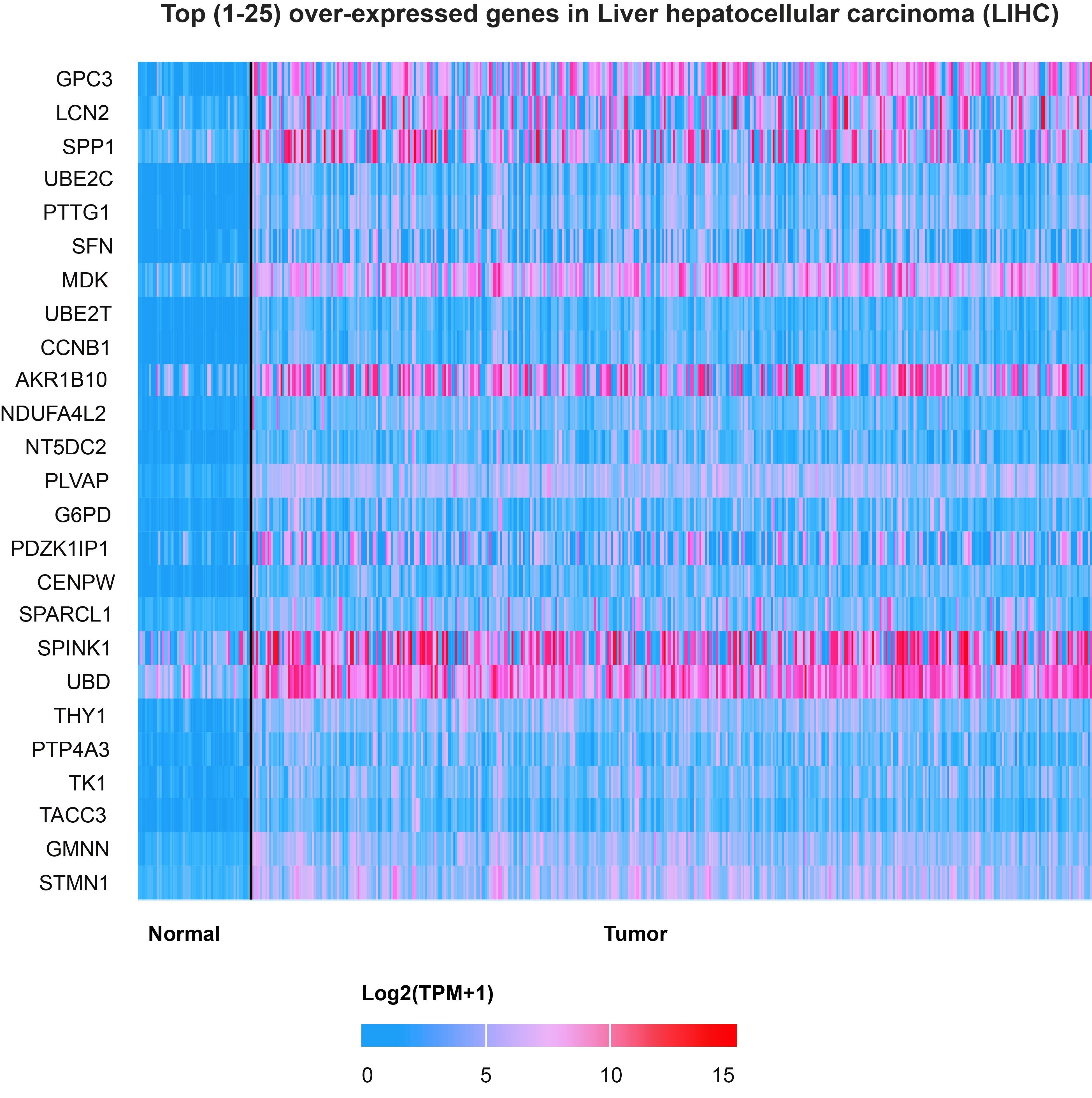


**Supplementary Fig. 3.** Discrimination and calibration of HCC prediction models through transcriptomics analyses in a training data set containing 1884 stage I-IV samples. (A) Receiving Operating Characteristic Curves (ROC) and Area Under the Curves (AUC) in models of SPINK1, SPINK1 plus AFP and AFP. (B) Calibration curve of AFP. (C) Calibration curve of SPINK1. (D) Calibration curve of SPINK1 plus AFP. (E) Clinical net benefits shown by DCA curves of SPINK1, SPINK1 plus AFP , and AFP. (F-H) Comparison in clinical benefits obtained from CIC analysis of each detection model as mentioned above.


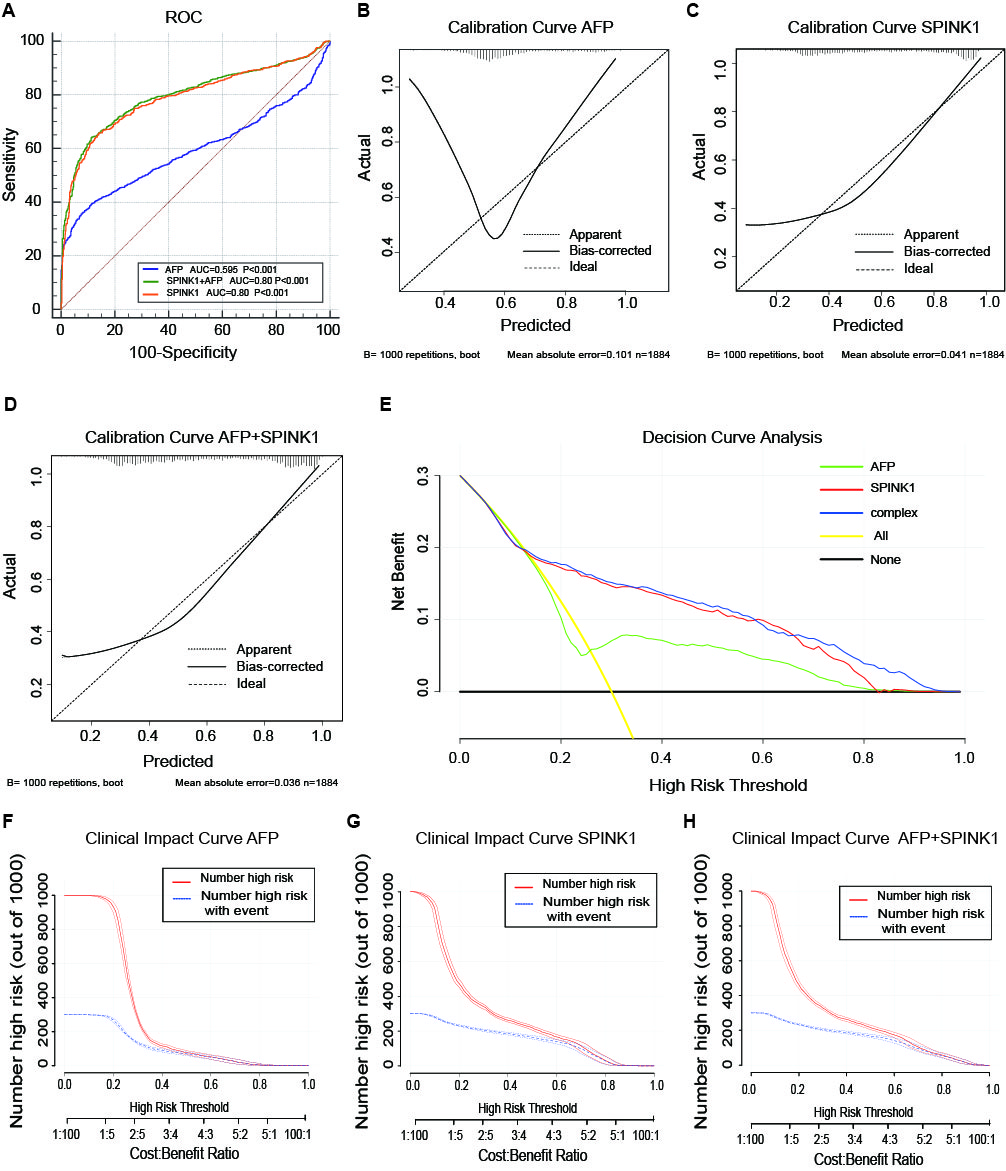


**Supplementary Fig. 4.** Correlation of SPINK1 and AFP in transcriptomics analyses and proteomics analyses.


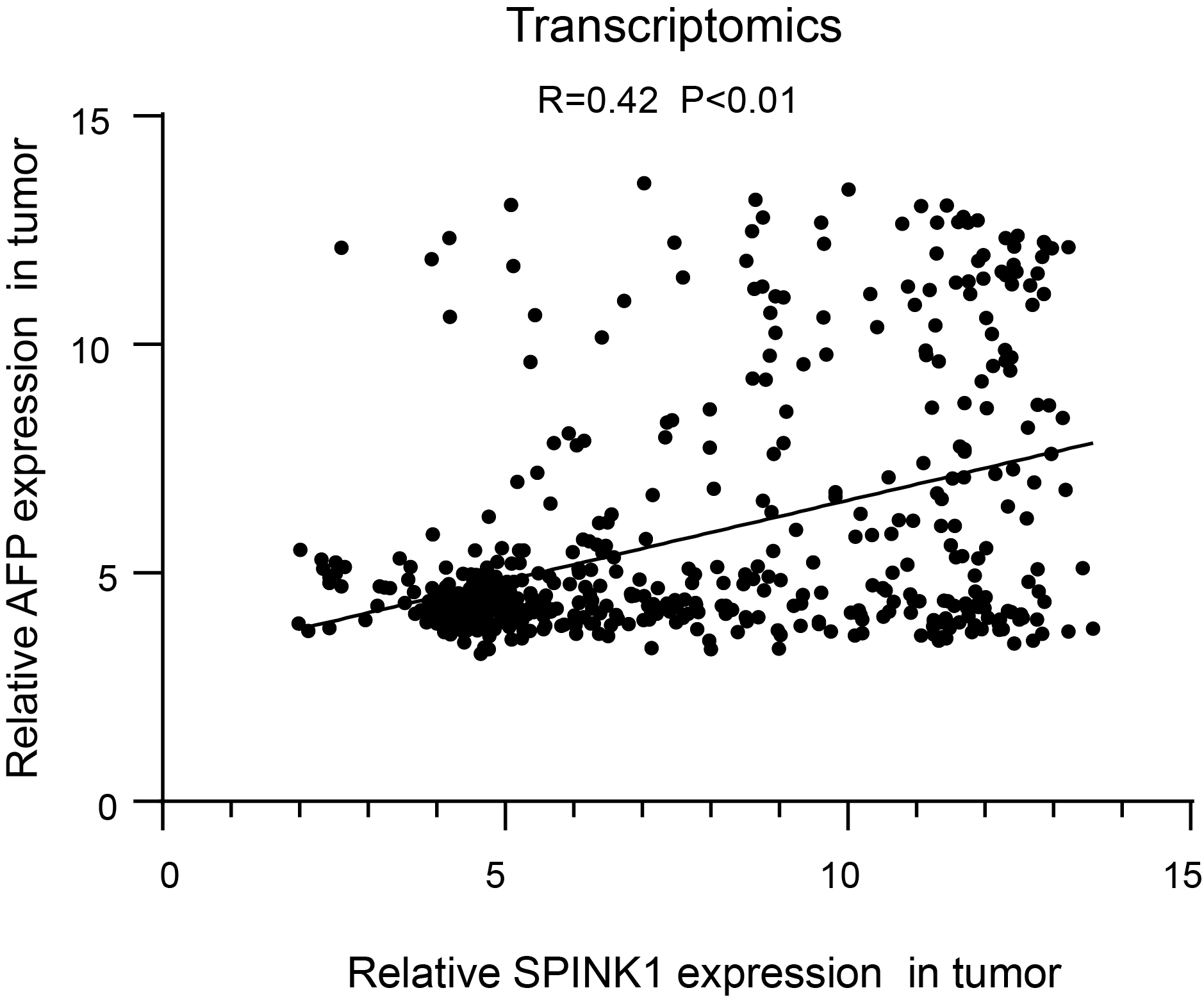


**Supplementary Fig. 5.** Discrimination and calibration of HCC prediction models through transcriptomics analyses in a validation data set No.2 (A) Receiving Operating Characteristic Curves (ROC) and Area Under the Curves (AUC) in models of SPINK1, SPINK1 plus AFP and AFP. (B) Calibration curve of AFP. (C) Calibration curve of SPINK1. (D) Calibration curve of SPINK1 plus AFP. (E) Clinical net benefits shown by DCA curves of SPINK1, SPINK1 plus AFP , and AFP. (F-H) Comparison in clinical benefits obtained from CIC analysis of each detection model as mentioned above.

**
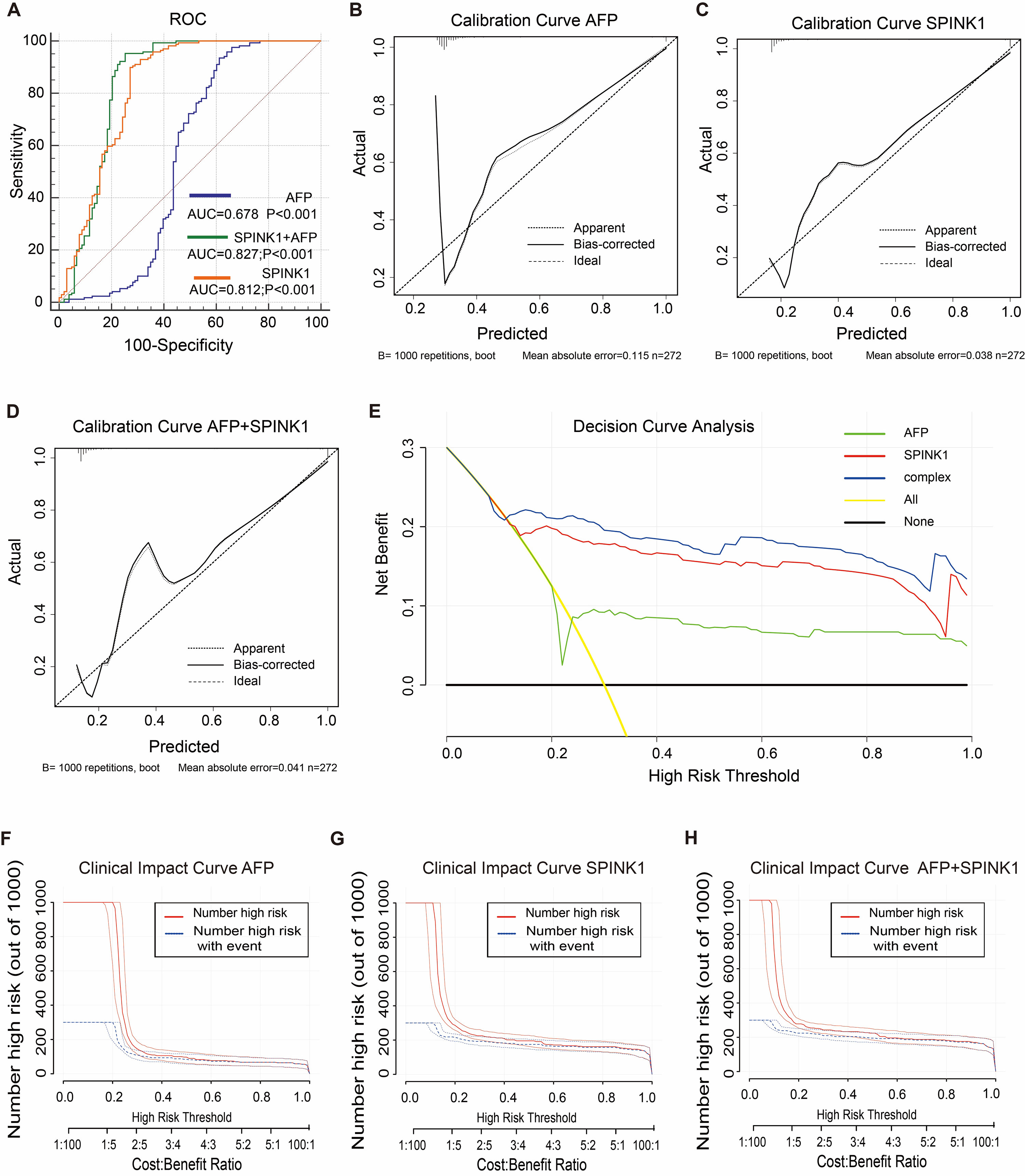
**

**Supplementary Fig. 6.** Correlation of SPINK1 and AFP in transcriptomics analyses and proteomics analyses.

**
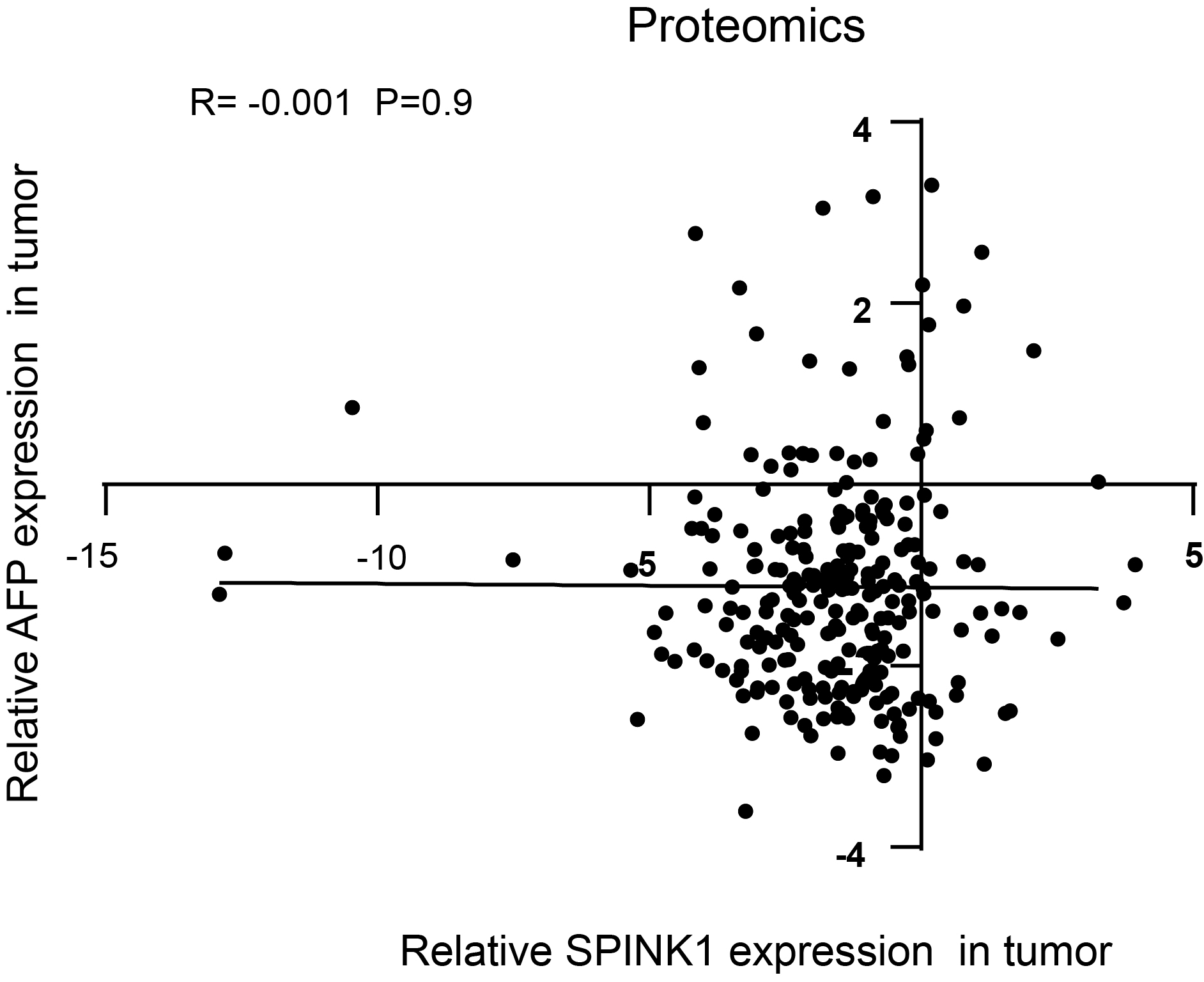
**

**Supplementary Fig.7.** Identification of lentivirally-transduced SPINK1 hepatocellular carcinoma (HCC) cells.


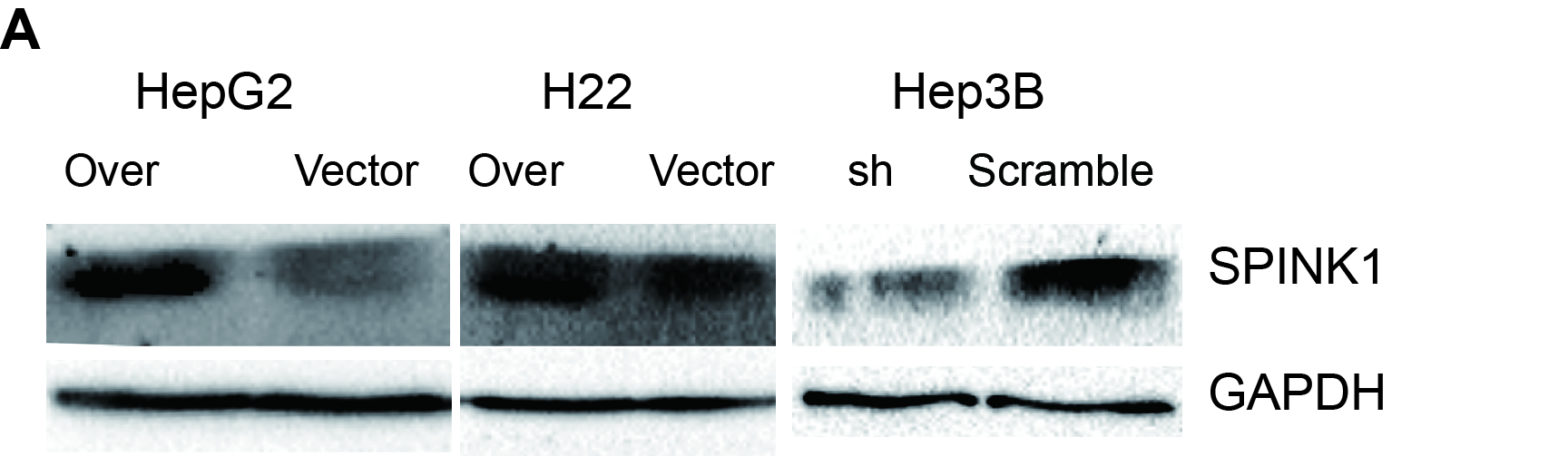


**Supplementary Fig. 8.** Compositions and contents of 28 subtypes of infiltrating immune cells in tissue samples of HCC patients from TCGA database

**
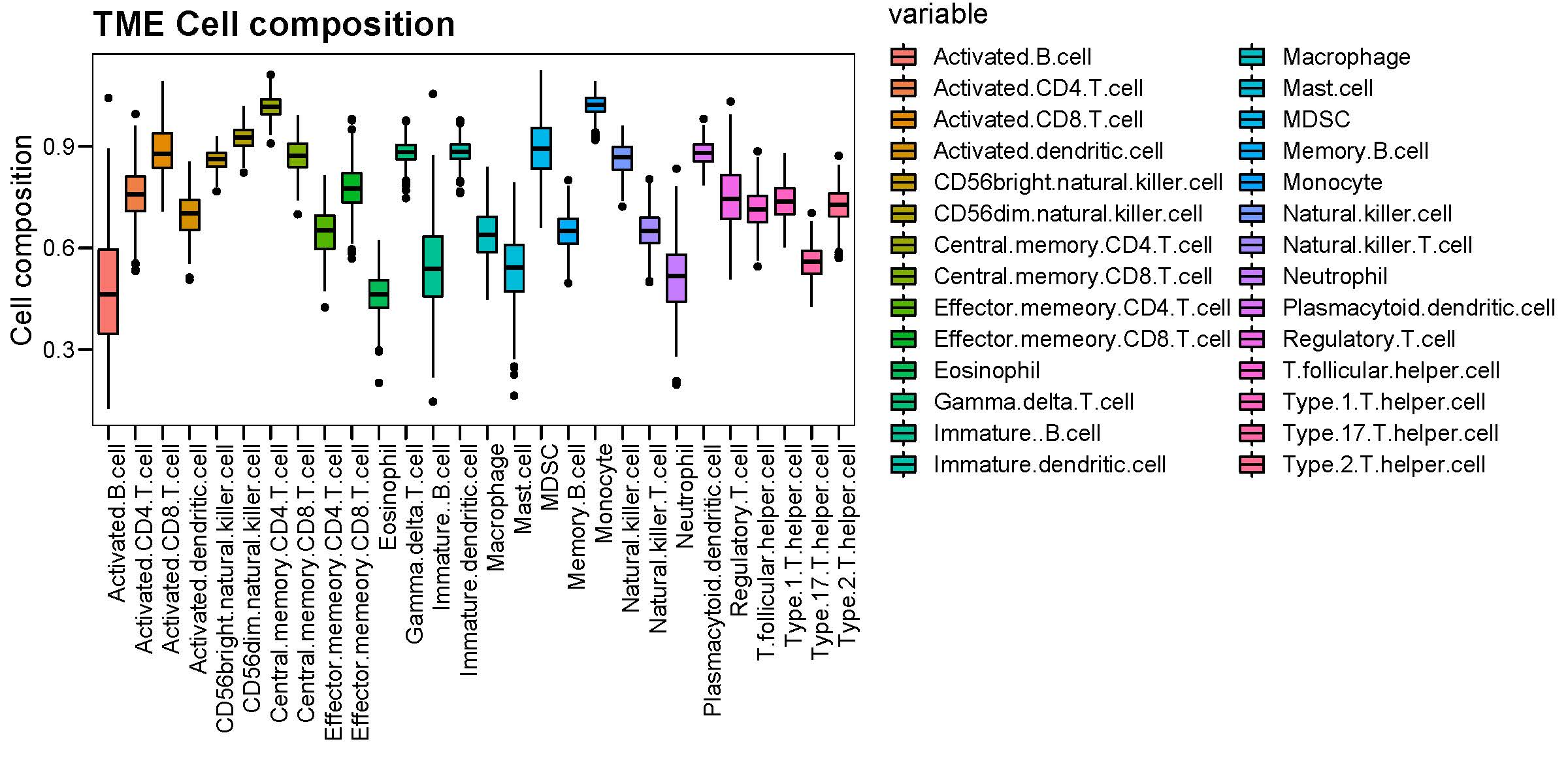
**

**Supplementary Fig. 9.** Correlations of SPINK1 transcriptomic expression with tumor mutation burden in HCC tissue specimens obtained from TCGA dataset. Analyses and comparison of tumor mutation burden (TMB) between SPINK1 expression -low and -high groups. P value 0.62 was obtained using statistical T test analysis. *, P < 0.05; **, P < 0.01; ***, P < 0.001.


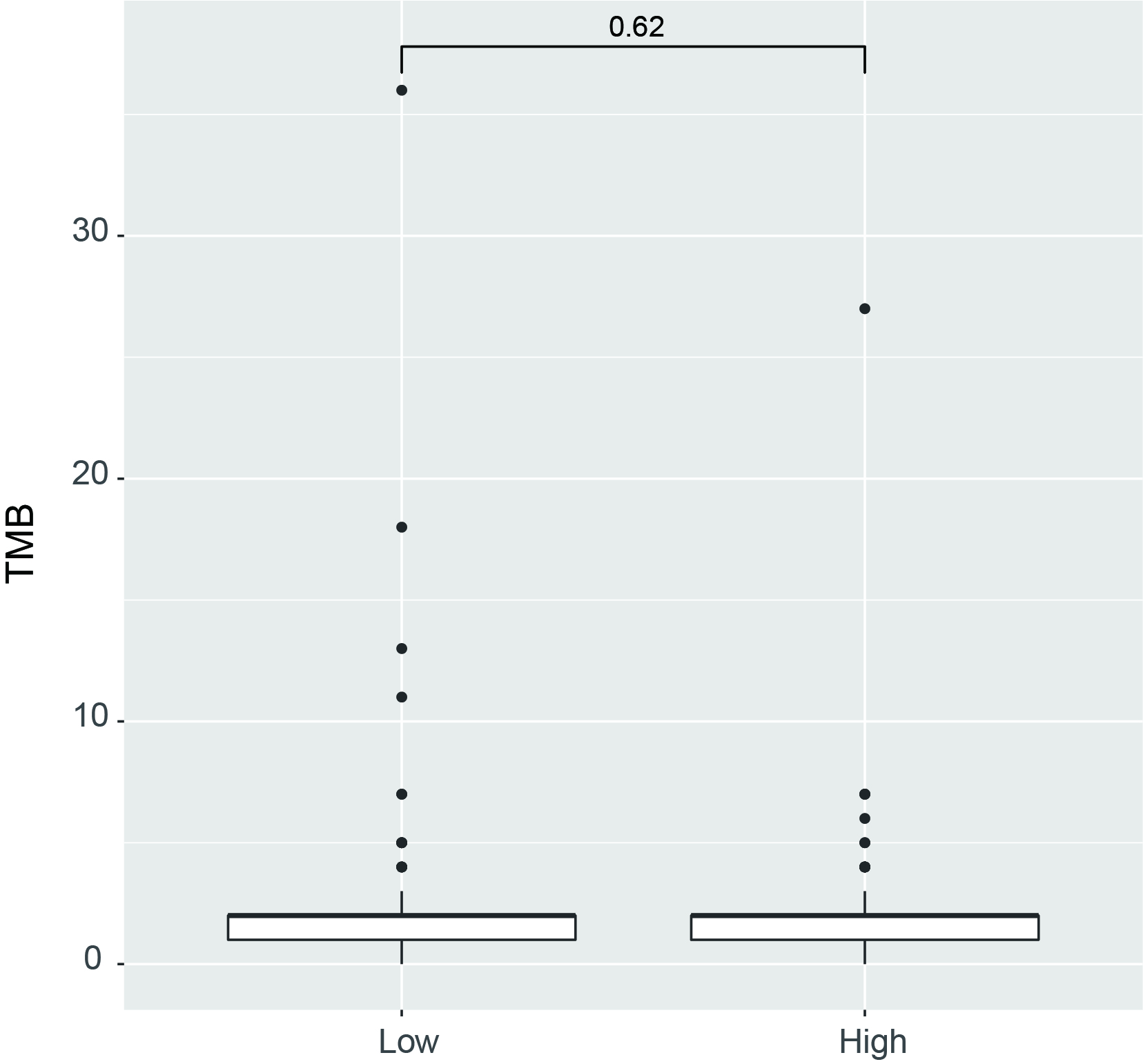

Supplement: Supplementary file 1 [file DataSheet_1.doc]
